# Spatial scale affects novel and disappeared climate change projections in Alaska

**DOI:** 10.1002/ece3.5511

**Published:** 2019-09-30

**Authors:** Bailey D. Morrison, Katy Heath, Jonathan A. Greenberg

**Affiliations:** ^1^ Environmental and Climate Sciences Department Brookhaven National Laboratory Upton New York; ^2^ Department of Plant Biology University of Illinois at Urbana‐Champaign Urbana Illinois; ^3^ Department of Natural Resources and Environmental Science University of Nevada, Reno Reno Nevada

**Keywords:** Alaska, climate change, climate downscale, last glacial maximum, novel and disappeared climates, spatial resolution

## Abstract

The formation of novel and disappeared climates between the last glacial maximum (LGM) and the present is important to consider to understand the expansion and contraction of species niches and distributions, as well as the formation and loss of communities and ecological interactions over time. Our choice in climate data resolution has the potential to complicate predictions of the ecological impacts of climate change, since climate varies from local to global scales and this spatial variation is reflected in climate data. To address this issue, we downscaled LGM and modern (1975–2005) 30‐year averaged climate data to 60‐m resolution for the entire state of Alaska for 10 different climate variables, and then upsampled each variable to coarser resolutions (60 m to 12 km). We modeled the distributions of novel and disappeared climates to evaluate the locations and fractional area of novel and disappeared climates for each of our climate variables and resolutions. Generally, novel and disappeared climates were located in southern Alaska, although there were cases where some disappeared climates existed within coastal and interior Alaska. Climate resolution affected the fractional area of novel and disappeared climates in three patterns: As the spatial resolution of climate became coarser, the fractional area of novel and disappeared climates (a) increased, (b) decreased, or (c) had no explainable relationship. Overall, we found the use of coarser climate data increased the fractional area of novel and disappeared climates due to decreased environmental variability and removal of climate extremes. Our results reinforce the importance of downscaling coarse climate data and suggest that studies analyzing the effects of climate change on ecosystems may overestimate or underestimate their conclusions when utilizing coarse climate data.

## Graphical abstract

We modeled the distributions of novel and disappeared climates to evaluate the locations and fractional area of novel and disappeared climates for multiple climate variables and resolutions. Climate resolution affected the fractional area of novel and disappeared climates in three patterns: As the spatial resolution of climate became coarser, the fractional area of novel and disappeared climates (a) increased, (b) decreased, or (c) had no explainable relationship. Our results reinforce the importance of downscaling coarse climate data and suggest that studies analyzing the effects of climate change on ecosystems may overestimate or underestimate their conclusions when utilizing coarse climate data.

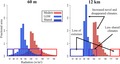

## 1 INTRODUCTION

Postglacial climate change provides a useful context and natural experimentation for assessing biotic responses to global climate change (Davis, 1990; Overpeck, Bartlein, & Webb, 1991; Webb III, 1992). The late quaternary, which includes the last glacial maximum (LGM) 21,000 years ago, matches the magnitude of predicted anthropogenic climate change and contains the largest manifestation of natural climate change preserved in the geologic record (Mix, Bard, & Schneider, 2001; Overpeck et al., 1991). Our awareness and understanding of past ecological responses to climate change is important as it enables ecologists to predict the potential responses of ecosystems to anthropogenic climate change now and in the future.

Niche theory predicts that *n*‐dimensional changes in the environment (e.g., precipitation and temperature) will cause shifts in species distributions and the formation of novel species assemblages, since every species responds individualistically to its abiotic and biotic environment (Hutchinson, 1957; Jackson & Overpeck, 2000). This assumption has been supported by large changes in species ranges where past climates lacked modern analogs, leading to the formation of novel LGM species associations and biomes with no modern equivalent (Williams & Jackson, 2007). Therefore, variations of climate in space and time (e.g., LGM vs. Modern *n*‐dimensional environment) are thought to be an important factor in understanding the formation of novel and disappeared climates, as well as the expansion and contraction of a species' niche and distribution (Ackerly et al., 2010; Jackson & Overpeck, 2000; Williams & Jackson, 2007). Novel climates are climatic environments with no‐analog conditions in the past, whereas disappeared climates are climatic environments with no‐analog conditions today (Ackerly et al., 2010; Chen, Hill, Ohlemuller, Roy, & Thomas, 2011; Fitzpatrick & Hargrove, 2009; Glassberg, 2014; Hobbs et al., 2006; Radeloff et al., 2015; Williams & Jackson, 2007).

There are many ways climate data can be used to describe shifts in climate, with the most extreme description being the detection of no‐analog climates. No‐analog climate techniques identify a location or period the climate of which is dissimilar to that in another context (Ford et al., 2010), and then classify the climate into spatial and/or temporal categories (Mearns et al., 2001). For example, spatial and temporal classifications of no‐analog climates typically describe local climate change using a dissimilarity metric (Grenier, Parent, Huard, Anctil, & Chaumont, 2013; e.g., Standardized Euclidean Distance) at the same grid cell between climate variables at two difference time periods, and then compare the climate realization for each land gridpoint from one time period the climate realization of all land gridpoints of the other time period, while retaining the minimum dissimilarity metric value (Ackerly et al., 2010; Fordham, Saltré, Brown, Mellin, & Wigley, 2018; Grenier et al., 2013; Ordonez, Williams, & Svenning, 2016; Williams, Jackson, & Kutzbacht, 2007). Other approaches have computed no‐analog climates relying only on temporal classifications of novel and disappeared climates. For example, Fitzpatrick and Hargrove (2009) utilized techniques employed by species distribution models to identify no‐analog climates in the future (areas where the current climate conditions of a study area do not exist in projected future climate), Burrows et al. (2014) calculated shifting climates using climate velocity (Burrows et al., 2014; Dobrowski et al., 2013; Loarie et al., 2009) to identify source (novel) and sink (disappeared) areas, and Wiens, Seavy, and Jongsomjit (2011) employed PCA analysis to collapse multidimensional climate into a single climate space to derive polygons encompassing current and future climate space to identify persisting climates, disappearing climates, and novel climates.

A key aspect of quantifying climate, including the identification of novel and disappeared climates, is the choice of spatial resolution. Spatial resolution has the potential to complicate the prediction of ecological impacts of climate change because climate varies dramatically at local scales and this variation is undetectable in coarse resolution climate data (Bellard, Bertelsmeier, Leadley, Thuiller, & Courchamp, 2012; Dobrowski, Abatzoglou, Greenberg, & Schladow, 2009; Franklin et al., 2013; Seo, Thorne, Hannah, & Thuiller, 2009). Long‐term climate patterns observed across the globe are a result of a combination of many different processes that occur at varying spatial scales. Most modeled climate data (regional and general circulation models) are coarse scale (>50 km), and these datasets are unlikely to incorporate many spatial features known to influence climate at finer scales (e.g., elevation gradients, coastal effects, temperature inversions, and rain shadows; Daly, 2006; Dobrowski et al., 2009; Levin, 1992).

Spatial variability in climate can be nested into macroclimates (global), mesoclimates (regional), topoclimates (landscape), and microclimates (local; Ackerly et al., 2010; Geiger, Aron, & Todhunter, 2009). Macroclimates are the broad patterns of atmospheric circulation across >50 km scales, such as the North–South rainfall gradient along the state of Alaska (Ackerly et al., 2010). Mesoclimates are variations at 1–50 km reflecting marine air and mountain range properties, such as rain shadow effects (Ackerly et al., 2010). Topoclimates include landscape scale effects such as aspect, slope, elevation, and terrain that affect surface radiation, wind, and cold‐air drainage at the 10‐m to 1‐km scale (Ackerly et al., 2010). Lastly, microclimates have the finest scale variability and are determined by vegetation cover and fine‐scale surface features (<10 m; Ackerly et al., 2010). Finer spatial scales (topo and micro) create unique combinations of climate variables within a very limited area (Ackerly et al., 2010) and can provide significant processes that buffer against larger regional and global climate trends (e.g., temperature inversions; Randin et al., 2009; Willis & Bhagwat, 2009). For example, mesoscale PRISM mean global temperature variability is limited to 3°C, while a finer toposcale mean global temperature surface at 30 m found a global temperature variability as high as 8°C (Ackerly et al., 2010; Daly, 2006).

We selected climate surfaces commonly used in climate change and ecological analyses to identify and determine where novel, disappeared, and shared climates are distributed across the state of Alaska from the LGM to modern era. Previous studies have identified multivariate no‐analog climates; however, we compute univariate no‐analog climates as species have been found to be limited by a single environmental factor (Liebig's Law; De Baar, 1994; Berryman, 2003; Crimmins, Dobrowski, Greenberg, Abatzoglou, & Mynsberge, 2011), and we could identify which specific aspects of multivariate climate cause no‐analog climates. Polar regions are currently experiencing the highest rates of warming (Larsen et al., 2014), making the state of Alaska, USA, an ideal location to study the impacts of spatial scale in climate change. To help clarify the effect of climate grid resolution on estimations of novel and disappeared climates, we analyzed how the amount of fractional area of novel, disappeared, and shared climates vary with climate grid spatial resolution by statistically downscaling coarse‐scale GCM (General Circulation Model) climate (~100 km) at scales ranging from 60 m to 12 km. We compared modeled distributions and fractional area of novel, disappeared, and shared climates across nine Alaskan ecoregions to ask the following specific questions:

1. Where are novel, disappeared, and shared climates located in Alaska from the LGM to modern era?
2. How does the fractional area of novel, disappeared, and shared climates differ depending on the resolution of modeled climate grid data used for analysis?

## 2 METHODS

### 2.1 Overview

The first step of our analysis was to perform a topographically mediated downscaling of coarse‐scale modern and LGM climate surfaces to a resolution of 60 m for the modern and LGM periods. Next, we upsampled these surfaces to coarser resolutions. Finally, we performed our analysis investigating the impacts of scale on the distribution of shared, novel, and disappeared climates throughout Alaska.

#### 2.1.1 Study area

Alaska, USA, is an ideal location for understanding the potential impacts of scale on climate change as polar regions currently experience the highest rates of warming globally, and by the end of the 21st century will be at least 40% higher than the global mean (Larsen et al., 2014). There is agreement between several coupled atmosphere–ice–ocean climate models that global warming should be enhanced in the Arctic (Raisanen, 2001). Additionally, Alaska has a diverse and complex physiography and presence of long‐term meteorological stations.

The total land area in Alaska is approximately 151,773.3 km^2^, with over 54,563 km of tidal shoreline, including islands, and stretches in latitude by nearly 20°. Seventeen of the 20 highest mountain peaks of North America are in Alaska, with the highest elevation at Mt. McKinley (6,150 m.a.s.l), and lowest at the Pacific Ocean coastline (0 m.a.s.l). Presently, there are approximately 100,000 glaciers covering 75,109.9 km^2^ (5% of the total land area of Alaska); however, during the LGM, glaciers covered an estimated 30% of the state. Present mean temperature is 16.8°C during the summer, and −11.0°C during the winter.

### 2.2 Climate downscale

#### 2.2.1 Input data

##### Weather station data

Spatially explicit monthly weather station data were collected from the National Oceanic and Atmospheric Administration's (NOAA) Global Historical Climatology Network (GHCND; Menne, Durre, Vose, Gleason, & Houston, 2012). We compiled records from 415 stations across the period of 1975–2005, although the period of record for each station varies. Missing data fields as well as extreme outliers were removed from the weather dataset by identifying observations >3 standard deviations from the means of all observations across time for minimum temperature (*T*_min_), maximum temperature (*T*_max_), mean temperature (*T*_ave_), and total monthly precipitation (*P*) (Aggarwal, 2016).

##### Digital elevation map and transforms

An elevation surface was assembled for the entire state of Alaska using U.S. Geologic Survey (USGS) 1 arc‐second (~60 m) digital elevation maps (DEM; USGS, 2016). The digital elevation map (DEM) was resampled and reprojected to Alaska Albers Equal Area Conic, ensuring grid cells of exactly 60 m. From this dataset, we calculated slope, aspect, and the topographic convergence index (TCI) for Alaska (Wolock & McCabe, 1995). TCI was calculated with TauDEM using a D‐infinity flow accumulation algorithm (Tesfa et al., 2011). Regions where slope was 0° were given a placeholder value of 0.001 when calculating TCI to avoid divide‐by‐zero issues.

##### General circulation model climate data

Coarse‐scale (1°) general circulation model (GCM) climate grids were collected from the National Center for Atmospheric Research's (NCAR) Community Climate System Model version 4 (CCSM4) for near surface (2 m) *T*_min_, *T*_max_, *T*_ave_, precipitation, short wave radiation (*I*_cloud_), and wind (U, V; Gent et al., 2011; Kluzek, 2011). All climate surfaces were pooled into two 30‐year averages, ~18,000–18,030 ya (years ago; *t*_1_) for the LGM and 1975–2005 (*t*_2_) for modern climate. All GCM surfaces were reprojected to the Alaska Albers Equal Area Conic projection and resampled to 60‐m resolution using bilinear interpolation. The wind speed vector surfaces were converted to wind speed and direction.

#### 2.2.2 Downscaling models

##### Shortwave irradiance

Solar radiation can regulate temperature in complex terrains as topography produces varying solar angles and can reduce terrain winds that diminish boundary layer mixing during the winter months (Daly, 2006; Dobrowski et al., 2009; Urban, Miller, Halpin, & Stephenson, 2000). Therefore, solar radiation is an important physiographic parameter to consider when downscaling temperature to topo and microscales. Mean monthly daily clear‐sky irradiance at a location *x*,*y* (*I*_(_ *_x_*_,_ *_y_*_,_ *_t_*_)_, W/m^2^) was modeled using the r.sun algorithm (Suri & Hofierka, 2004) running under GRASS GIS 7.1. This algorithm uses topographic elevation, slope, aspect, and geographic latitude as inputs referenced against solar angles. GRASS GIS's r.sun algorithm is a complex and flexible solar radiation model that has been found to outperform other similar products (SolarFlux, Solei, Solar Analyst, and SRAD) because it performs especially well for large areas at fine resolutions with complex terrain and can be used for long‐term calculations at different scales (Hofierka & Suri, 2002; Ruiz‐Arias, Tovar‐Pescador, Pozo‐Vázquez, & Alsamamra, 2009). To create “true sky” clouded irradiance surfaces for both the LGM and today, we acquired coarse modeled surface downwelling shortwave radiation (100 km) that was calculated by the Coupled Model Intercomparison Project (CMIP5, Modern) the Paleoclimate Modeling Intercomparison Project (PMIP5, LGM; Taylor, Stouffer, & Meehl, 2012). These groups calculate surface downwelling shortwave radiation using additional modeled climate surfaces produced from the same CCSM4 LGM and modern historical runs used in our temperature and precipitation downscale models. To transform our high‐resolution “clear‐sky” irradiance to “true‐sky” irradiance, we calculated the ratio of true‐sky to clear‐sky irradiance for each month for both the LGM and today, and then applied each corresponding ratio to each monthly high‐resolution clear‐sky irradiance surface to create twelve monthly LGM and Modern “true‐sky” surface radiation surfaces that are capable of considering coarse atmospheric properties (e.g., cloud cover) of each era with our clear‐sky radiation surfaces. We then summarized our monthly radiation surfaces into an annual average radiation surface to compute novel and disappeared radiation climates for later analysis. Mean radiation was created by computing the yearly average of all monthly radiation surfaces for both the LGM and modern eras at 60‐m resolution.

##### Temperature and precipitation

For minimum, maximum, and average temperature, as well as precipitation, we used an empirical downscaling approach as described in Dobrowski et al. (2009). This approach calibrates a downscaling model in which the high‐resolution climate variable is a function of the coarse‐scale climate and various topographic predictors (Equation 1). We used the following general downscaling approach:

(1) $$\text{Observed}\;{\text{Climate}}_{x,y,t}^{\text{High}\;\text{Res}}=f(\text{CCSM}\;{\text{Climate}}_{x,y,t}^{\text{Coarse}\;\text{Res}}+{\text{Physiographic}}_{x,y,t}^{\text{High}\;\text{Res}}$$

where observed Climate at location (*x,y*) at time *t* represents our high‐resolution weather station data. CCSM Climate at the same (*x,y*) locations and time* t* represents our coarse GCM modeled climate. Physiographic Inputs at the same (*x,y*) locations, and when applicable time *t*, represents our high resolution modeled physiographic surfaces (e.g., elevation, cold‐air pooling, and radiation). We assume the topographic impacts on climate do not change in time, only in space, so the only varying predictors in our model are coarse‐scale climate and radiation inputs. Our other topographic‐based predictors remain constant (e.g., TCI).

We calibrated these models using weather station data to represent the high‐resolution climate linked with the GCM climate grids that correspond to the date of the weather measurement. To determine variable selection, we used a random forest algorithm to derive variable importance statistics for all model parameters at a variety of spatial scales (60 m, 500 m, 1 km, and 5 km; Breiman, 2001). Using the most important variables, we produced three models for temperature (*T*_min_, *T*_max_, *T*_ave_) calibrated using 80% of the available weather station data (43,768 observations; Madsen & Thyregod, 2010). The remaining 20% of weather station data (10,943 observations) was used for model validation, in which we calculated the root mean square error (RMSE), Pearson's correlation coefficient, and percent bias. We created a precipitation model employing similar methods previously used to create our temperature models; however, the precipitation model was trained using a stratified sampling procedure (vs. 80% dataset) to ensure an equal proportion of measured weather station precipitation values were represented in the training dataset since the majority of weather observations recorded 0 mm/month precipitation. The training dataset was stratified in ~310 mm width bins from randomly sampling 80% of the original precipitation dataset. We then randomly sampled 5,000 precipitation values from each bin to create the final training dataset. Our testing dataset was comprised from the remaining 20% of the original precipitation dataset.

Generalized linear models (GLM) were chosen to generate all downscale models. GLMs are advantageous for downscaling because they can extrapolate beyond the range of the model's training data, which is necessary when predicting LGM climates as the range of climates is likely different than the modern climate. Additionally, GLMs can handle more complicated situations; for example, they do not require a normal distribution of the response variable (Madsen & Thyregod, 2010). A variety of tests/exploratory models were created to check that all GLM assumptions were met. To ensure that all input predictors were independent of one another, all variables were plotted against one another to verify that there were no significant relationships between each variable. A correlation threshold of 0.95 was used as a cutoff value preventing too great a multicollinearity between any pair of predictors. Additionally, an exploratory generalized additive model (GAM) was created to produce partial plots for each independent variable to verify linear behavior of all model inputs, although this method was not chosen for the final models to avoid spline interpolations on our predictors (Venables & Ripley, 2002). Lastly, each model's residuals were examined to ensure that the residuals were normally distributed. The residuals were plotted against the predicted fitted values to ensure a homogenous structure of each model's variance.

##### Temperature model

The model we used to downscale *T*_min_, *T*_max_, and *T*_ave_ was found to be a function of elevation at *x,y* (*Z*_(_ *_x,y_*_)_), mean monthly daily clear‐sky irradiance at a location *x,y* (*I*_(_ *_x,y,t_*_)_), and TCI (*C*_(_ *_x,y_*_)_) which is a proxy for local convective forcing's such as cold‐air pooling (Equation 2; Dobrowski et al., 2009; Katurji & Zhong, 2012). The final model form was

(2) $$T_{x,y,t,t^{\prime }}=T_{x,y,t,t^{\prime }}^{\prime }+Z_{\left(x,y\right)}+C_{\left(x,y\right)}+I_{\left(x,y,t,t^{\prime }\right)}+\varepsilon$$

where *T*_(_ *_x,y,t,t′_*_)_ represents observed high‐resolution temperature (min, max, average) at a given station (*x,y*) and month (*t*), and *T′*_(_ *_x,y,t,t′_*_)_ is coarse modeled surface temperature (min, max, average). The coldest *T*_min_ month (December for LGM, and January for modern) was used to represent the coldest annual temperatures. Likewise, the hottest month for *T*_max_ (July for both LGM and modern) was used to represent the hottest annual temperatures.

##### Precipitation model

The model to downscale precipitation was found to require topographic predictors at a variety of scales, not just the high resolution 60 m predictors. Specifically, we used elevation at location *x,y* (*Z*_(_ *_x,y_*_)_) at 60 m; topographic slope at location *x*,*y* at 1‐km resolution (*m_x,y_*); wind speed (*m*/*s*, _(_ *_x,y,t,t_*_′)_) and wind direction at a given location (*x,y*) (*degrees*, _(_ *_x,y,t,t_*_′)_) and month (*t*) at 100 km; and the angular difference between geographic direction and topographic aspect to act as an orographic effect proxy (i.e., rain shadow) at 500‐m resolution (*_x,y,t,t_*_′_). Elevation and slope exhibited slight nonlinear trends which were corrected by applying a log‐transformation to each of the two predictor variables. A cubic root transformation was applied to (*P_x,y,t,t_*_′_) and (*P*′*_x,y,t,t_*_′_) so the model could not predict values below 0 mm/month (Equation 3). The final model form was

(3) $${\left(P_{x,y,t,t^{\prime }}\right)}^{1/3}={\left(P_{x,y,t,t^{\prime }}^{\prime }\right)}^{1/3}+\text{log}\left(Z_{x,y}\right)+\text{log}\left(m_{x,y}\right)+\omega_{x,y,t,t^{\prime }}+\theta_{x,y,t,t^{\prime }}+\Delta_{x,y,t,t^{\prime }}+\varepsilon$$

##### Potential evapotranspiration

Plants must use energy and water to grow and reproduce; therefore, the primary effects of climate on plants are regulated by the interactions of energy and water (Stephenson, 1990). Through water balance equations, energy is represented by potential evapotranspiration (PET) and available water (Stephenson, 1998). PET was calculated at monthly time steps at 60 m for the LGM and modern era using the Penman–Monteith method which utilizes our downscaled temperature, elevation, wind speed, and cloud‐corrected irradiance surfaces as inputs (Allen, Pereira, Raes, & Smith, 1998; Monteith, 1965; Penman, 1948). This method determines a reference evapotranspiration from climate data and represents climatic water balance rather than physiological evapotranspiration. PET was calculated by using a standard hypothetical reference crop of height 0.12 m with a fixed surface stomatal resistance of 70 s/m and albedo of 0.23. Annual PET for the LGM and modern era was summarized by computing the summation of all monthly surfaces for each era.

##### Actual evapotranspiration and water deficit

The interactions of PET and available water can be described with additional water balance parameters: actual evapotranspiration (AET) and water deficit (DEF). AET and water deficit are biologically meaningful parameters that are well correlated with the distribution of vegetation types compared to other parameters such as temperature and precipitation (Stephenson, 1998). PET (i.e., evaporative demand) represents the total amount of energy available in the environment; essentially, the evaporative water loss from a site with unlimited water and is used to derive estimates of AET and water deficit (Stephenson, 1990). AET is the evaporative water loss given the actual water availability at a site and therefore represents the biologically usable energy and water in the environment (Stephenson, 1990). Water deficit refers to climatic water deficit, not soil water deficit, and represents the amount of evaporative demand that was not met by available water in other words: WD = PET − AET (Stephenson, 1990). While AET and water deficit are important for understanding the climatic controls of vegetation distributions, it is worth mentioning that all three parameters (PET, AET, and water deficit) are climatic products representing available water and energy at a site, not biological products dependent of specific vegetation types (Stephenson, 1998).

AET and DEF were calculated at monthly time scales at 60 m for the LGM and modern era, by using a snow hydrology model that models additional water and energy parameters that influence available water at a site (Dobrowski et al., 2013). Downscaled precipitation, mean temperature, and radiation were used to estimate the fraction of precipitation arising as either rainfall, snowfall, snowpack, or snowmelt during a given month at 60‐m resolution for both the LGM and modern eras (Lutz, Van Wagtendonk & Franklin, 2010; Dobrowski et al., 2013). These products were then used to calculate AET and DEF in combination with PET and a coarse soil water‐holding capacity surface (Dunne & Willmott, 1996) to determine the available plant extractable water from rainfall or snowmelt from the previous month (Dobrowski et al., 2013). A spatially constant available soil water‐holding capacity (AWC) value of 5.0 cm (the mean AWC of Alaska) was used rather than a spatially varying AWC due to a lack of high‐resolution AWC products in Alaska (Dunne & Willmott, 1996). The model first determines the amount of maximum potential available soil water at a site. If there is an excess of soil water, AET is the same as PET because evapotranspiration is not limited by water availability. However, if available water is less than the sites maximum potential available water, AET will be less than PET (AET = PET − DEF), because maximum water evaporative demand cannot be met by the available amount of water present at the site. We created annual summaries of each surface (AET, DEF, snow, and rain) by computing the summation all monthly surfaces, for each climate variable, for each individual era.

#### 2.2.3 Climate aggregation and reclassification

To understand how climate grid resolution can potentially affect the distribution of novel and disappeared climates in Alaska, we aggregated each 60 m annual climate surface to coarser resolutions. Aggregation surfaces were created using a standard average pixel aggregation of each 60 m annual climate surface using the following resolutions: 120, 240, 800 m (e.g., PRISM), 1 km (e.g., NASA Nex & WorldClim), 2, 3, 4, 5, 10, and 12 km (e.g., CMIP; Daly, Gibson, Doggett, Smith, & Taylor, 2004; Fick & Hijmans, 2017; Thrasher et al., 2013). Table 1 lists the final annual surfaces and grid units for our novel and disappeared climate analysis.

**Table 1:** Summary of annual downscaled (60 m) climates for the LGM and modern eras used in novel and disappeared climate analysis

Climate variable	Units	Min	Max	Mean	*SD*
LGM					
Annual AET	mm	0.0	651.7	184.9	88.1
Annual Deficit	mm	0.0	420.6	22.5	33.1
Annual PET	mm	0.3	667.4	207.4	93.9
Annual rain	mm	0.0	3,452.9	231.2	223.2
Annual snow	mm	0.2	5,494.4	782.2	439.2
Annual radiation	w	0.0	261.0	131.0	23.4
*T*_max_	°C	−21.0	30.5	16.9	8.1
*T*_min_	°C	−36.1	4.3	−21.9	3.4
Modern					
Annual AET	mm	0.0	445.4	183.5	49.0
Annual deficit	mm	0.0	314.4	11.5	15.0
Annual PET	mm	1.7	463.5	195.0	52.0
Annual rain	mm	0.0	4,424.7	505.6	330.0
Annual snow	mm	0.0	3,420.5	717.2	263.1
Annual radiation	w	0.0	208.7	97.8	17.1
*T*_max_	°C	−13.2	31.3	21.0	3.7
*T*_min_	°C	−34.7	6.6	−18.1	5.4

Abbreviations: AET, actual evapotranspiration; LGM, last glacial maximum; PET, potential evapotranspiration.

Annual climate variables at each of the 11 spatial resolutions were binned to simplify the ranges of possible climate values for analysis. We computed the 5th and 95th percentiles of each 60 m annual climate variable and removed these values as outliers in each surface. The range of each 60 m annual climate variable was determined and used to create 25 equal‐sized bins for each variable to reassign pixel values. The value of 25 bins was chosen to avoid empty bins within the range of the coarsest (12 km) surfaces for all climate variables. Bin widths were computed for each surface using the following equation:

(4) $$\text{Bin}\;\text{Width}=\frac{\left(\text{MaxRange}\left(\left.\text{LGM}\right|\text{Modern}\right)-\text{MinRange}\left(\left.\text{LGM}\right|\text{Modern}\right)\right)}{\text{Max}\;\text{Bin}\;\text{Value}}$$

where MaxRange(LGM||Modern) is the maximum value of between the LGM or modern climate surface at 60 m, MinRange(LGM||Modern) is the minimum value between the LGM or modern climate surface at 60 m, and Max Bin Value is the maximum bin class value (25). To reclassify each annual climate surface at all resolutions, the bin widths were applied to each LGM and modern climate surface using the following equation:

(5) $$\text{Bin}\;\text{Class}=\frac{\text{Ceiling}\left(\text{Climate}\;{\text{Value}}_{x,y,t,t^{\prime }}-\text{Min}\left(\text{Climate}\;{\text{Surface}}_{t}\right)\right)}{\text{Bin}\;\text{Width}}$$

where Climate Value*_x,y,t_* is the climate surface value at location *x,y* and at time *t* (LGM or modern), and Min(Climate Surface*_t_*) is the minimum value of the climate surface at time *t*. Pixels that fell within the removed 1st and 99th ranges were reclassified to either the lowest (0) or highest (25) bins for final reclassification.

**Figure 1:**
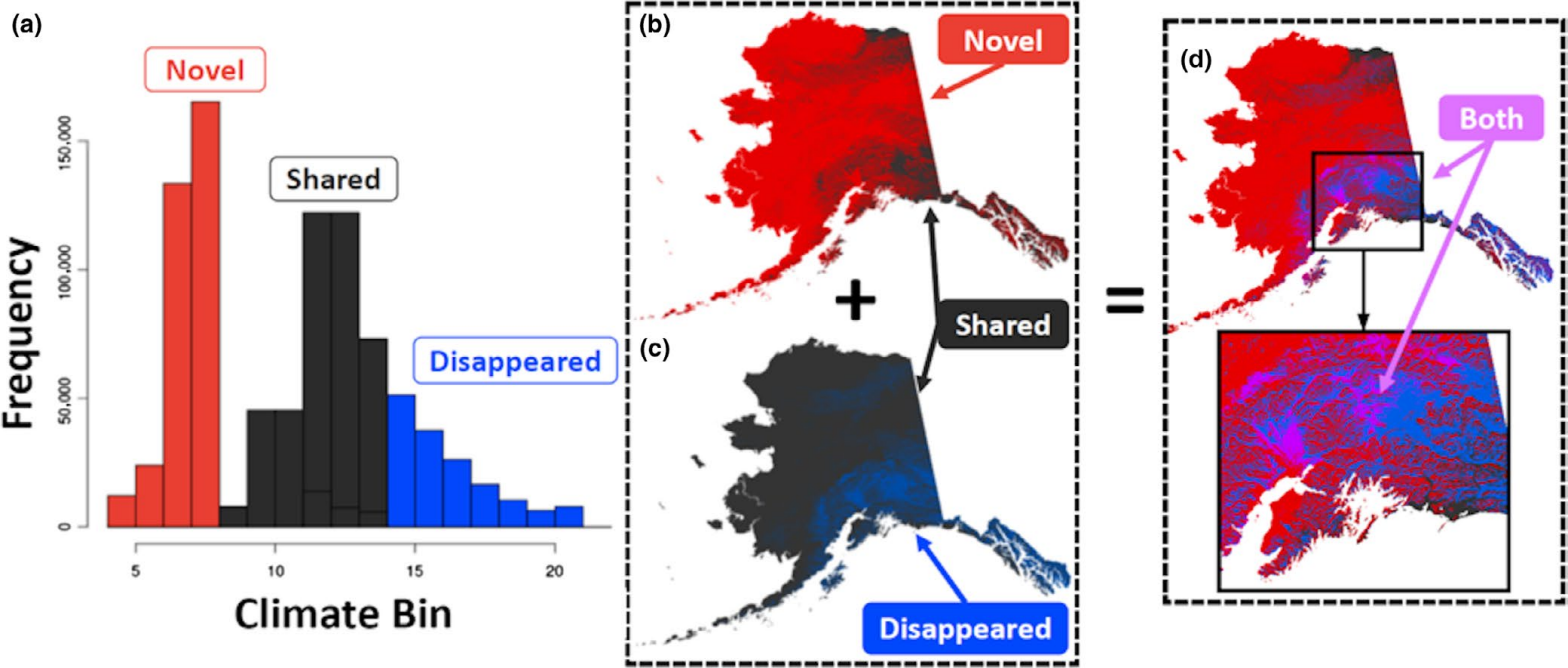
Classification process for “novel,” “disappeared,” “shared,” and “both” climates. 2 km climate surfaces were used for visualization purposes for this figure, as “both” climates are almost nonexistent at 60‐m resolution. Novel climates are red, disappeared climates are blue, shared climates are black, and both novel and disappeared climates are purple. (a) Histogram of LGM and Modern radiation climate bin ranges to define novel, disappeared, and shared climate bins. (b) Novel/Shared climate locations at 10 km. (c) Disappeared/Shared climate locations at 10 km. (d) Union of maps b and c. Purple pixels are intersection locations where climate was both novel and disappeared at 10 km

Novel and disappeared climate distributions for each climate surface were computed at all resolutions and eras from the reclassified climate surfaces. Novel climates were defined as modern climate bins that did not exist during the LGM and disappeared climates as LGM climate bins that did not exist in the modern period (Figure 1a). Using these definitions, we then determined which bins constituted novel, disappeared, or shared climates for each climate variable and era. Pixels for each climate surface, resolution, and era were reclassified to (a) novel, (b) disappeared, (c) shared, or (d) novel and disappeared climates based on each variable's novel and disappeared bin definitions. If a specific climate existed only during the LGM, the climate range was considered “disappeared” (blue pixels; Figure 1c). If a specific climate exists only in the modern era, the climate range was considered “novel” (red pixels; Figure 1b). If a specific climate existed during both time periods, the climate was considered “shared” (black pixels), as that specific range of climate could be found either during the LGM or modern era within the extent of Alaska (Figure 1b,c). Lastly, it is possible for a specific climate range to be both novel and disappeared at the same location. For instance, during the LGM, minimum temperatures at some locations were so cold they are not observed in the modern era. However, in the exact same location during the modern era, minimum temperatures may have warmed so much due to anthropogenic climate change that they were not present during the LGM. If a specific climate was both novel and disappeared at the same location, the climate range was considered “both” (purple pixels; Figure 1d).

The locations of novel and disappeared climates were summarized by computing the fractional area of novel, disappeared, and shared climates for the entire state of Alaska, as well as the fractional area among nine “Level 2” Alaskan ecoregions defined by the U.S. Geologic Survey (Gallant, Binnian, Omernik, & Shasby, 1995). We used modern AK ecoregions for both the modern and LGM eras, since, to the best of our knowledge, no LGM ecoregion boundary datasets currently exist. We quantified the amount of novel, disappeared, or shared climate at a given spatial resolution and used a Spearman's rank correlation coefficient to determine whether the relationship between novel, disappeared, or shared fractional climate and spatial resolution was positive, negative, or no apparent relationship.

## 3 RESULTS

### 3.1 Downscale model evaluations

**Figure 2:**
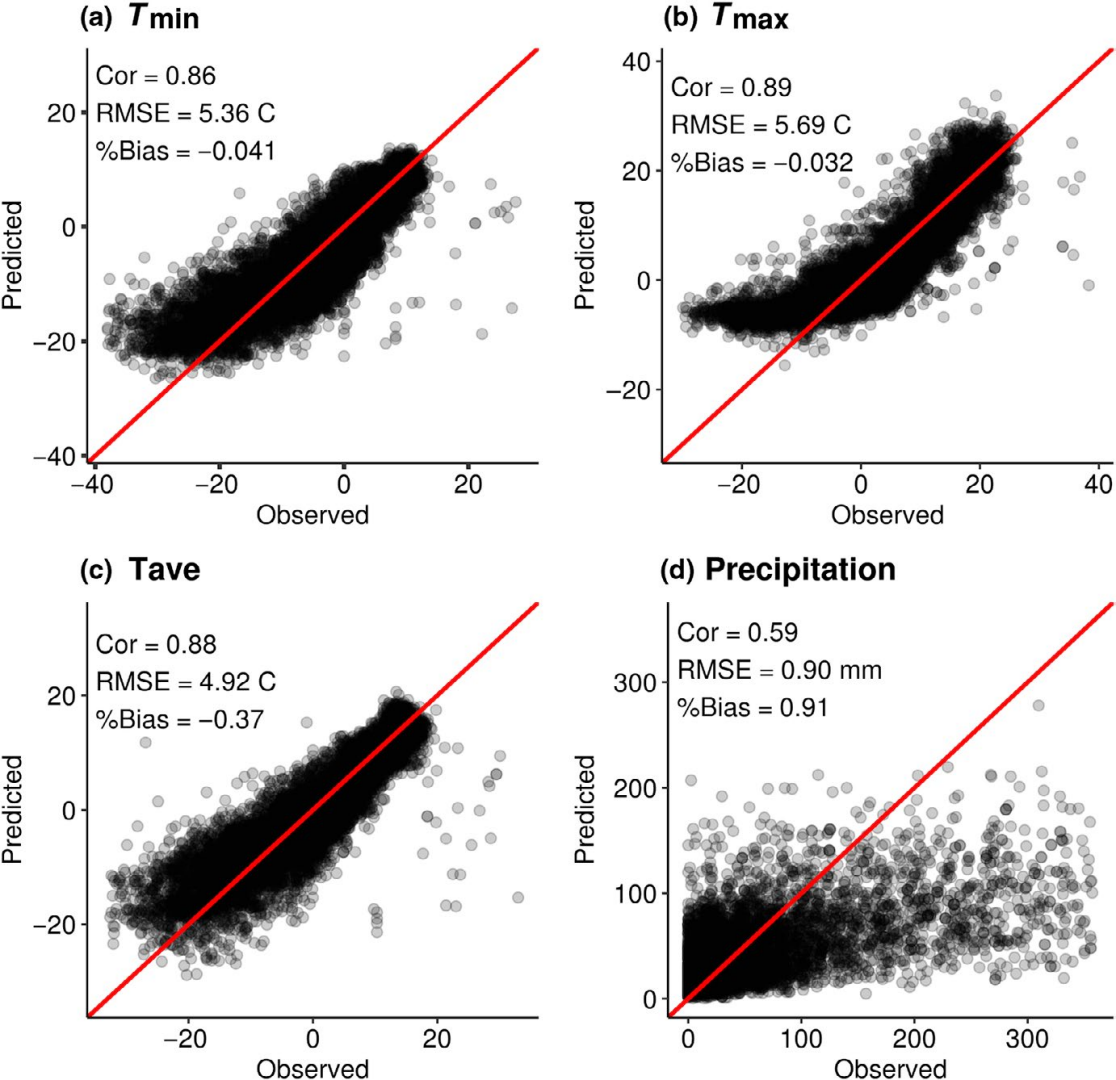
Downscale model evaluations. All plots are observed weather station data versus predicted downscaled climate values, with correlation, RMSE, % Bias, and a 1:1 red correlation line to represent at hypothetical perfect correlation between observed and predicted values. (a) *T*_min_ Model, (b) *T*_max_ Model, (c) *T*_ave_ Model, (d) Precipitation Model performance

**Figure 3:**
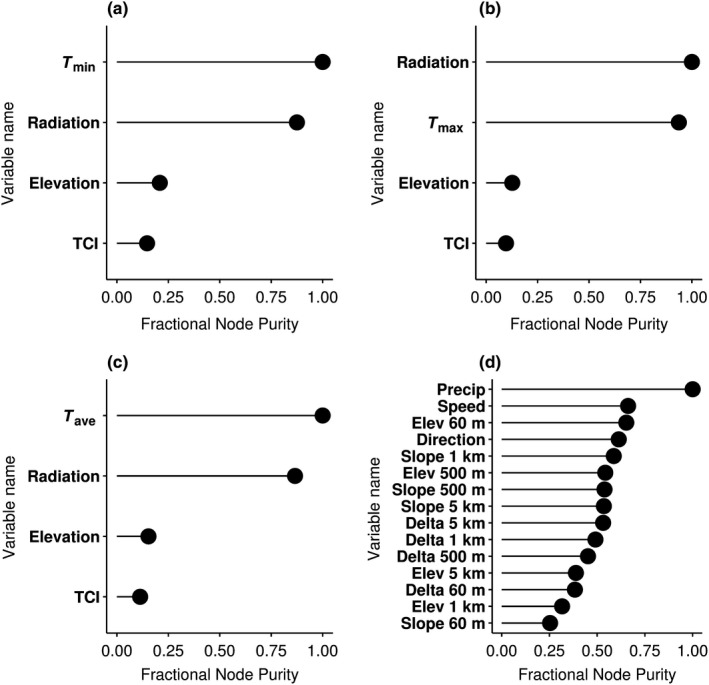
Variable importance plots for downscaled climate models based on random forest model preliminary investigations. (a) *T*_min_ Model, (b) *T*_max_ Model, (c) *T*_Ave_ Model, (d) Precipitation model. Bold precipitation model variables indicate variables selected from random forest importance and spatial resolution

All temperature model (*T*_min_, *T*_max_, *T*_ave_) assumptions of GLMs methods were met. Correlation coefficients (0.86, 0.86, 0.89), root mean square error (RMSE, 5.36, 5.96, 4.92°C), and percent bias (−0.041, −0.032, −0.37) for the final GLM for *T*_min_, *T*_max_, and *T*_ave_ models suggest that all models were well calibrated (Figure 2a–c). Independent variables (*T*_Coarse,_ elevation, TCI, and radiation) for all three temperature models had significant *p*‐values of <0.001, confirming the importance of all model predictors. In all three models, coarse modeled temperature, followed closely by radiation, was the most significant contributor to model performance. Elevation and TCI, while still important, were less significant as determined by exploratory random forest model variable importance plots (Figure 3a–c).

All model assumptions of the precipitation GLM were met. As expected, our precipitation model correlation was lower than our temperature downscale models, with a higher RMSE and percent bias, because downscaling procedures for modeling precipitation are less well understood than downscale temperature methods. The Pearson's correlation coefficient (0.59), RMSE (0.90 mm), and percent bias (0.91) for the precipitation model indicated that the basic GLM model was not as well calibrated (Figure 2d). However, the precipitation model's correlation falls within the range of reported correlation values from other previously downscale precipitation products, indicating that our precipitation model is acceptable given preceding precipitation model standards (Cannon, 2011; Maraun et al., 2010; Wetterhall, Bardossy, Chen, Halldin, & Xu, 2006; Widmann, Bretherton, & Salathé, 2003). Independent precipitation variables (*P*_coarse_, log(elevation), log(slope), wind direction, and orographic effects) had significant *p*‐values of <<.001, again, validating our choice in predictors. Wind speed was the least significant predictor for our precipitation model with a *p*‐value of .0961; however, wind speed was still somewhat beneficial in predicting precipitation patterns across Alaska. Overall, elevation was the most significant predictor of precipitation, followed by slope, coarse modeled precipitation, orographic effects, wind speed, and lastly, wind direction as the weakest predictor as indicated by our random forest variable importance plots (Figure 3d).

### 3.2 Downscaled novel and disappeared climate distributions

**Figure 4:**
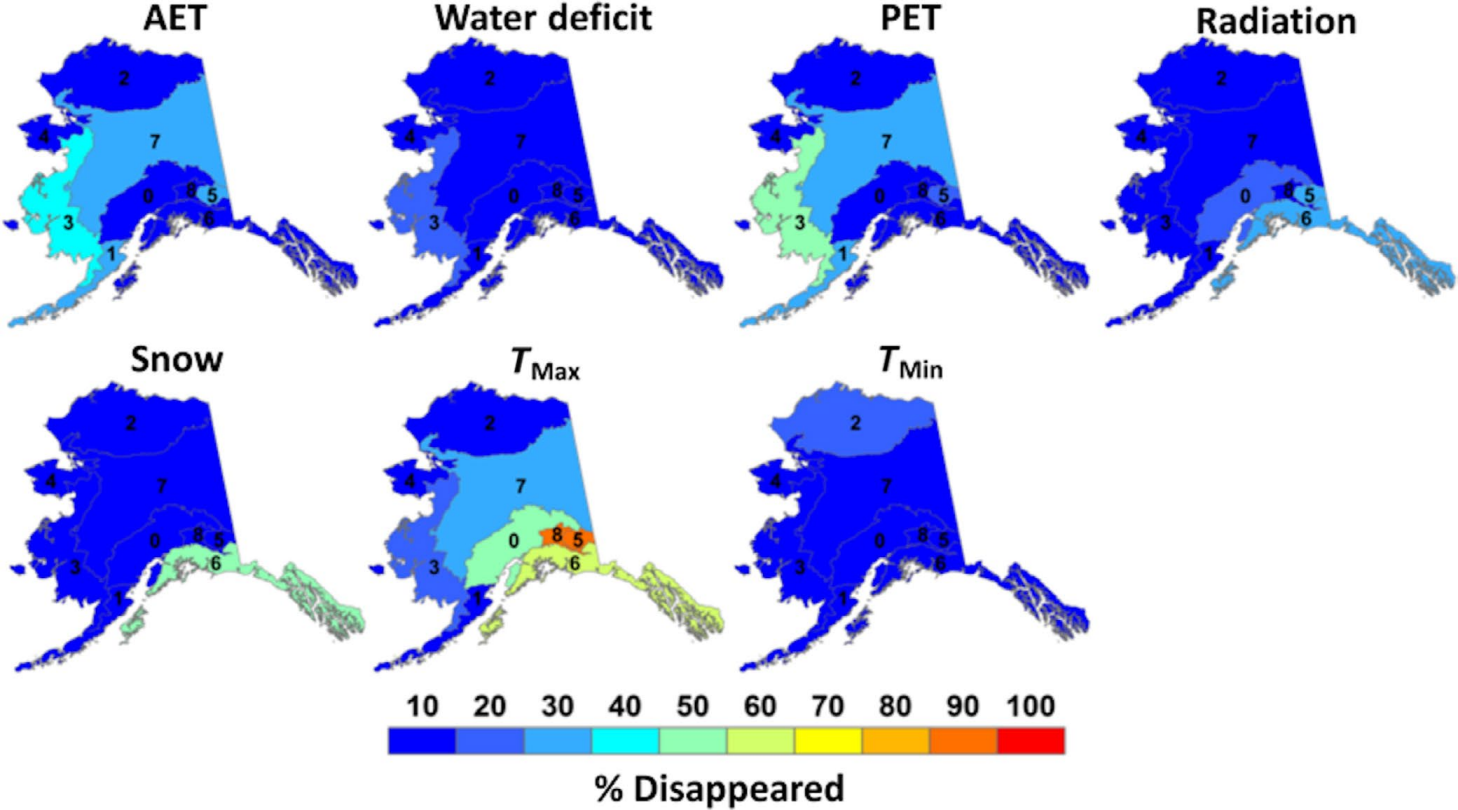
Fractional area coverage of disappeared climates for the nine “Level 2” Alaskan ecoregions. [1] Alaska Range Transition, [2] Aleutian Meadows, [3] Arctic Tundra, [4] Bering Taiga, [5] Bering Tundra, [6] Coast Mountain Transition, [7], Coastal Rainforests, [8] Intermontane Boreal, [9] Pacific Mountain Transition

Disappeared climates were defined as LGM climates that did not exist during the modern era. Seven of the eight downscaled climate surfaces contained disappeared climates across Alaska, with rain being the exception. Overall, disappeared climates covered 55.9% of Alaska from the LGM to modern era. *T*_max_ displayed the highest amount of disappeared climates in Alaska, covering 33.6% of the state alone, while water deficit displayed the lowest amount of disappeared climates at 2.9% (Table 2). For most ecoregions, disappeared climates covered <30% of Alaska, although >30% of some coastal ecoregions were covered by disappeared climates, especially for *T*_max_ (Figure 4). Ecoregions most affected by disappeared maximum temperature climates were the Coast Mountain Transition (85.0%) and Pacific Mountain Transition (82.5%). The Coastal Rainforest ecoregion displayed the largest amount of disappeared snow climates in the state, covering 40.0% of the region (Figure 4). Western Alaska also experienced disappeared climates in the Aleutian Meadows and Bering Taiga ecoregions due to disappeared PET climates; however, the fraction of disappeared climates were not as high as the southern portion of the state at 37.1 and 43.9%, respectively (Figure 4). The Bering Tundra ecoregion contained the least amount of disappeared climates for all climate variables, containing <10% of disappeared climates for the entire region. All other ecoregions and climate variables contained varying amounts of disappeared climates across Alaska (Figure 4).

**Table 2:** Percent cover (based on fractional area) for novel, disappeared, and shared climates for each climate variable in Alaska from the LGM to modern era

Type	AET	Water deficit	PET	Snow	*T*_max_	Rad	*T*_min_	Rain
% Novel	0.0	0.0	0.0	0.0	0.0	2.0	5.5	3.7
% Disappeared	15.6	2.9	15.6	6.0	33.6	13.0	6.3	0.0
% Shared	84.4	97.1	84.4	94.0	66.4	85.1	88.2	96.3
% Both	0.0	0.0	0.0	0.0	0.0	0.0	0.0	0.0

Abbreviations: AET, actual evapotranspiration; LGM, last glacial maximum; PET, potential evapotranspiration.

**Figure 5:**
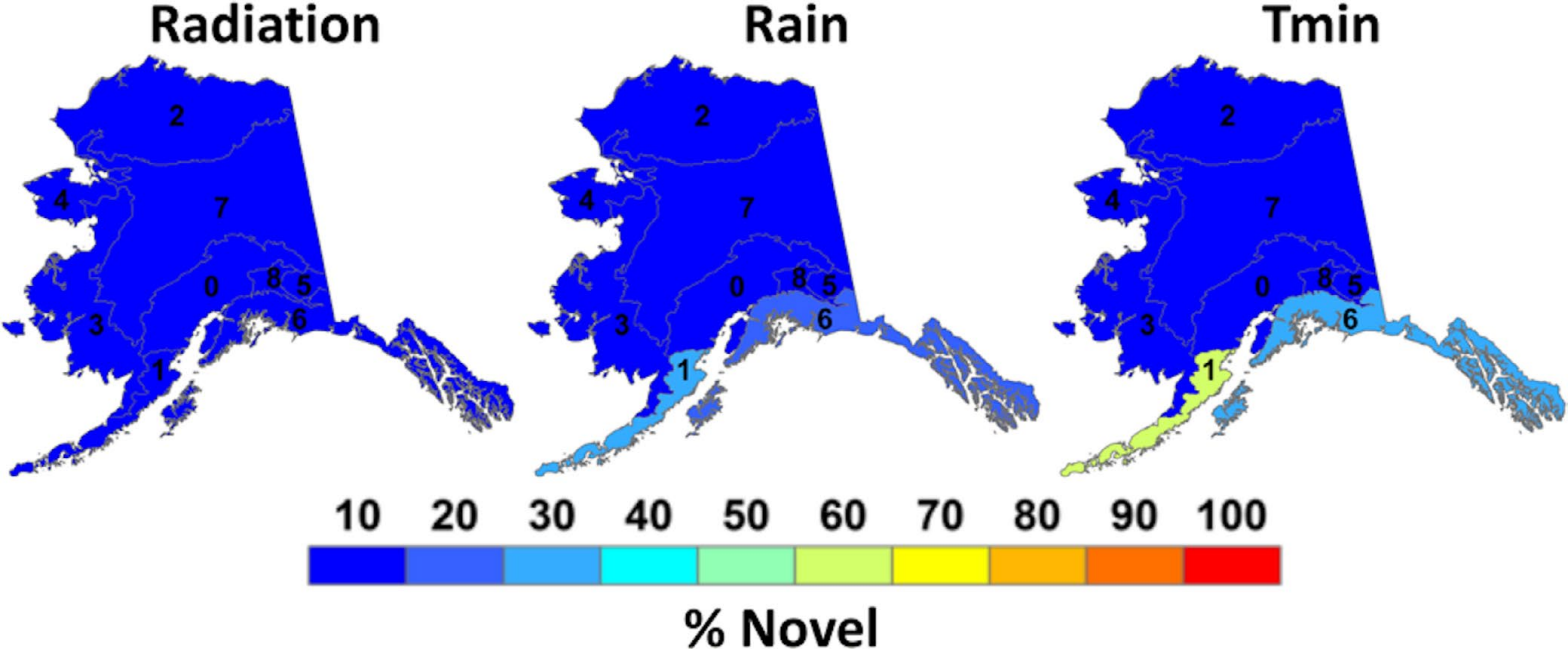
Fractional area coverage of novel climates for the nine “Level 2” Alaskan ecoregions. [1] Alaska Range Transition, [2] Aleutian Meadows, [3] Arctic Tundra, [4] Bering Taiga, [5] Bering Tundra, [6] Coast Mountain Transition, [7], Coastal Rainforests, [8] Intermontane Boreal, [9] Pacific Mountain Transition

Novel climates were defined as modern climates that did not exist during the LGM. Of the eight downscaled climate surfaces, only radiation, rain, and *T*_min_ contained novel climates in Alaska. Novel climates were less common than disappeared climates in Alaska, covering <10.0% across all of Alaska from the LGM to modern era. *T*_min_ displayed the highest amounts of novel climates in Alaska, covering 5.5% of the state, while radiation displayed the smallest amount of novel climates at 2.0% (Table 2). Most ecoregions were covered by <10% of novel climates for all three climate variables, except for the Aleutian Meadows and Coastal Rainforests ecoregions of Alaska from *T*_min_ climates (Figure 5). *T*_min_ contained the largest amounts of novel climates for both ecoregions, at 56.9% and 24.8%, respectively. While less than novel *T*_min_ climates, novel rain also affected these ecoregions covering 29.7% and 17.6%, respectively (Figure 5). Radiation contained some novel climates in Alaska; however, all ecoregions in Alaska had <4% novel radiation climates present in Alaska from the LGM to modern era (Figure 5).

**Figure 6:**
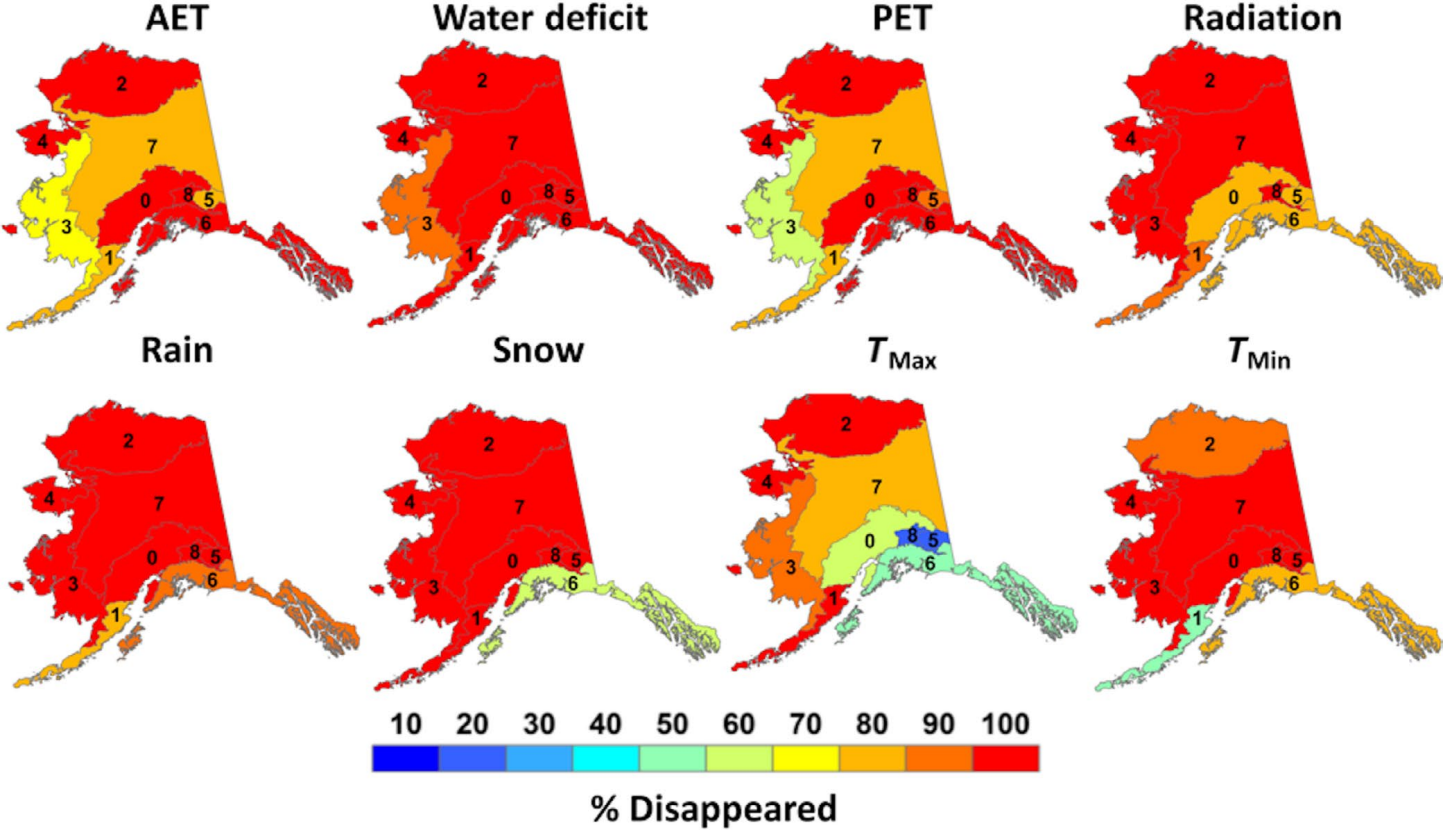
Fractional area coverage of shared climates for the nine “Level 2” Alaskan ecoregions. [1] Alaska Range Transition, [2] Aleutian Meadows, [3] Arctic Tundra, [4] Bering Taiga, [5] Bering Tundra, [6] Coast Mountain Transition, [7], Coastal Rainforests, [8] Intermontane Boreal, [9] Pacific Mountain Transition

Shared climates were defined as climates that existed during both the LGM and modern eras. As expected, all eight climate surfaces contained shared climates throughout Alaska, since no climate surface was classified as 100% novel or disappeared. Overall, shared climates generally covered more of Alaska than novel or disappeared climates, with a minimum fractional area of 40.6%. Shared water deficit climates covered 97.1% of the state, indicating water deficit climates have changed least in Alaska (Table 2). Additionally, shared *T*_max_ climates covered 66.4% of Alaska, indicating that *T*_max_ climates have changed the most in the state from the LGM to modern era (Table 2). This was expected as water deficit contained no novel climates and the lowest amount of disappeared climates, while *T*_max_ contained no novel climates, but the largest amount of disappeared climates in Alaska. While all Alaskan ecoregions contained various amounts of shared climates, there were isolated cases where ecoregions were <80% shared, although this was expected whenever high novel or disappeared climates covered the same ecoregion and climate variable (Figure 6). The Arctic Tundra, Bering Tundra, and Intermontane Boreal ecoregions contained the highest overall shared climate distributions in Alaska at 84.9%, 98.28%, and 75.3%, indicating that these ecoregions are generally less affected by postglacial climate change (Figure 6). The Coast and Pacific Mountain Transition ecoregions experienced the lowest amounts of shared climates at 15.0% and 17.5%, indicating that these ecoregions are generally more affected by postglacial climate change (Figure 6).

While it is possible for downscaled novel and disappeared climates to exist at the same location from the LGM to modern era, only a very small fraction of *T*_min_ climates displayed this phenomenon in Alaska at 60‐m resolution and was determined to be insignificant as it only covered <0.001% (39.6 km^2^) of Alaska. At coarser resolutions, there was a noticeable increase in climates classified as “both” novel and disappeared for some climate surfaces at a location; however, this portion of the results concentrates on our high‐resolution downscaled climate surfaces (“both” climates vs. spatial resolution is discussed in the next section).

### 3.3 No‐analog climates versus spatial resolution

**Figure 7:**
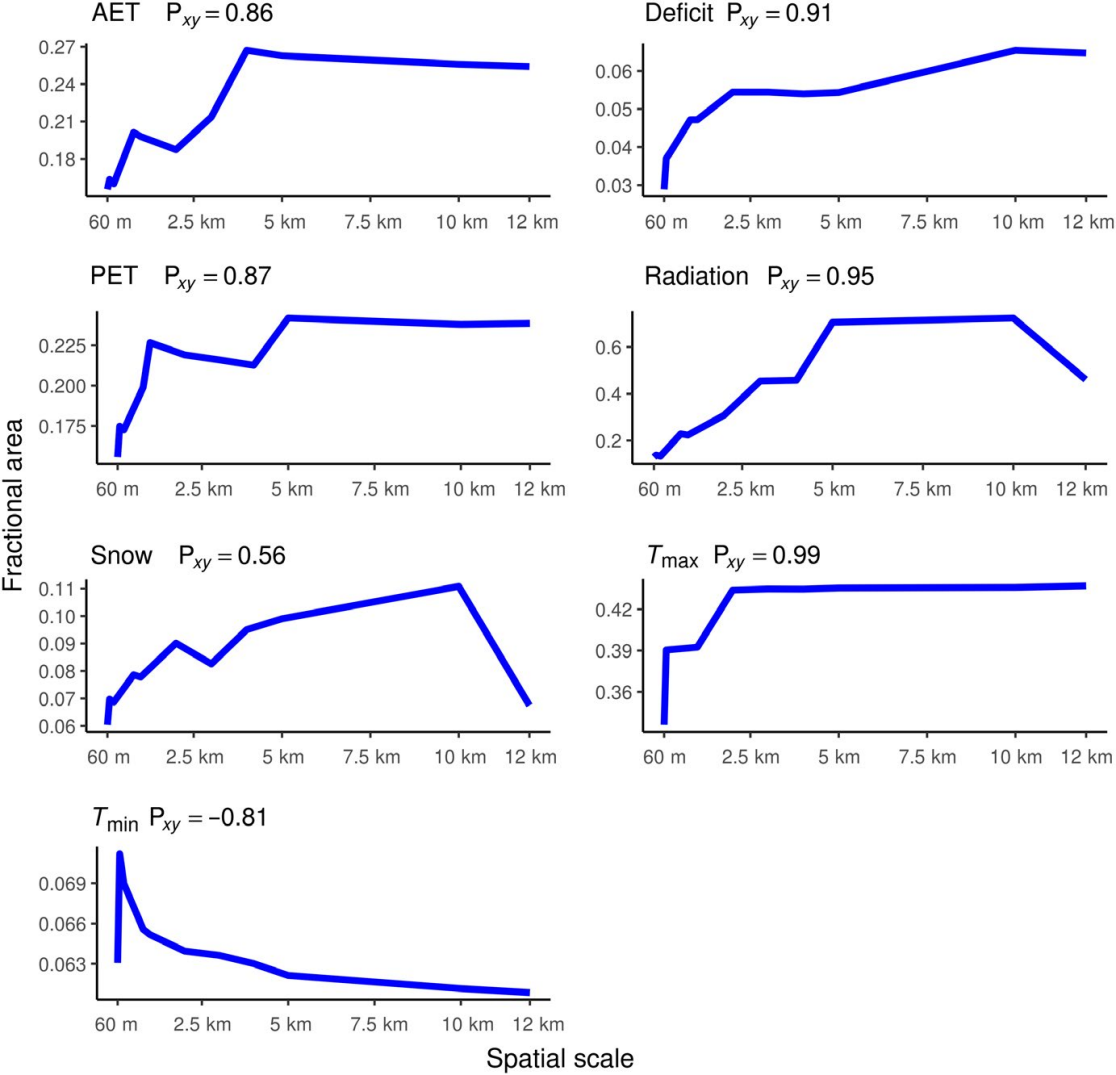
Disappeared climate versus spatial scale plots. Spatial scale (60 m–12 km) plotted against fractional area of disappeared climates for each climate variable. The Spearman's rank correlation coefficient is included in each plot

**Figure 8:**
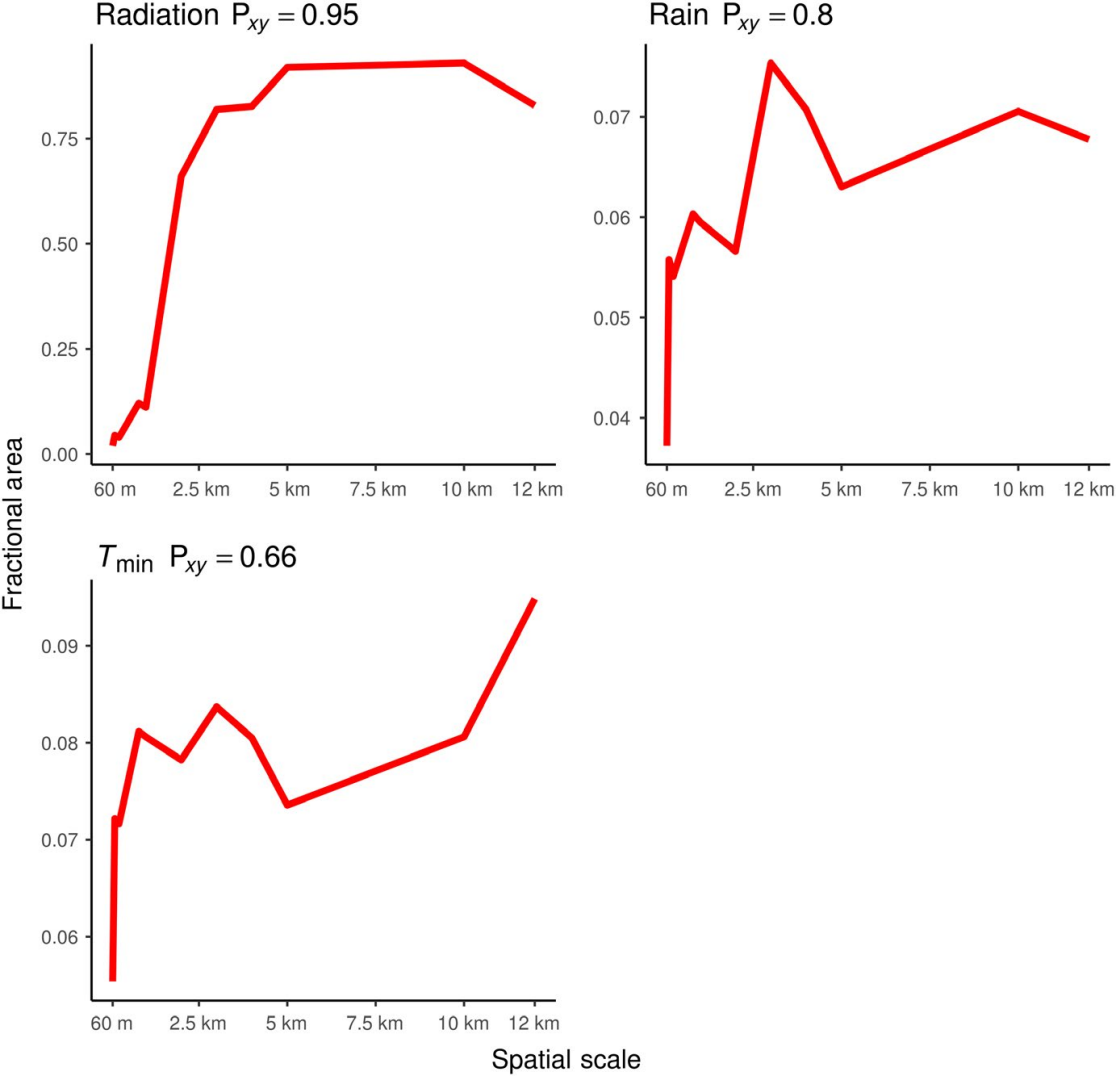
Novel climate versus spatial scale plots. Spatial scale (60 m–12 km) plotted against fractional area of novel climates for each climate variable. The Spearman's rank correlation coefficient is included in each plot

**Figure 9:**
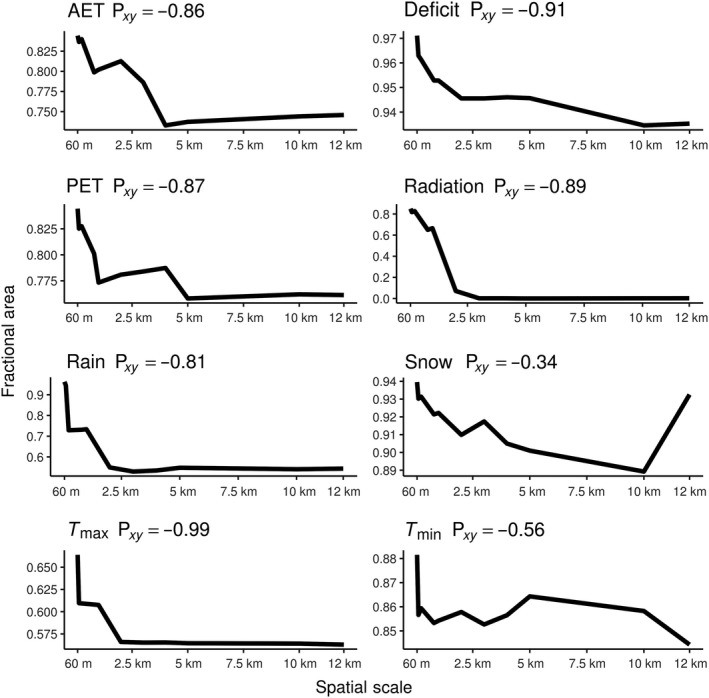
Shared climate versus spatial scale plots. Spatial scale (60 m–12 km) plotted against fractional area of shared climates for each climate variable. The Spearman's rank correlation coefficient is included in each plot

As spatial resolution becomes coarser, the fractional area of novel and disappeared climates generally increases, while the fractional area of shared climates generally decreases. All disappeared climate surfaces, except minimum temperature, had moderate (0.40–0.59) to very strong (0.80–1.00) Spearman's rank correlations between spatial resolution and fractional area, indicating that as the spatial resolution of climate becomes coarse, the fractional area of disappeared climates increases (Figure 7). All novel climate surfaces had a strong (0.60–0.79) to very strong (0.80–1.00) Spearman's rank correlation coefficient between spatial resolution and fractional area, indicating that as spatial resolution of climate becomes coarser, the fractional area of novel climates increases as well (Figure 8). All climate variables had a negative relationship between fractional area of shared climate and spatial resolution, as expected. The strengths of Spearman's rank correlations varied from moderate (−0.40 to 0.59) to very strong (−0.80 to −1.00; Figure 9).

An exception to the previous patterns, disappeared *T*_min_, produced a very strong (0.80–1.0) negative Spearman's rank correlation of 0.81 (Figure 7). The negative relationship of this correlation coefficient suggests that as spatial resolution becomes coarser, the amount of fractional disappeared *T*_min_ climates decreases.

**Figure 10:**
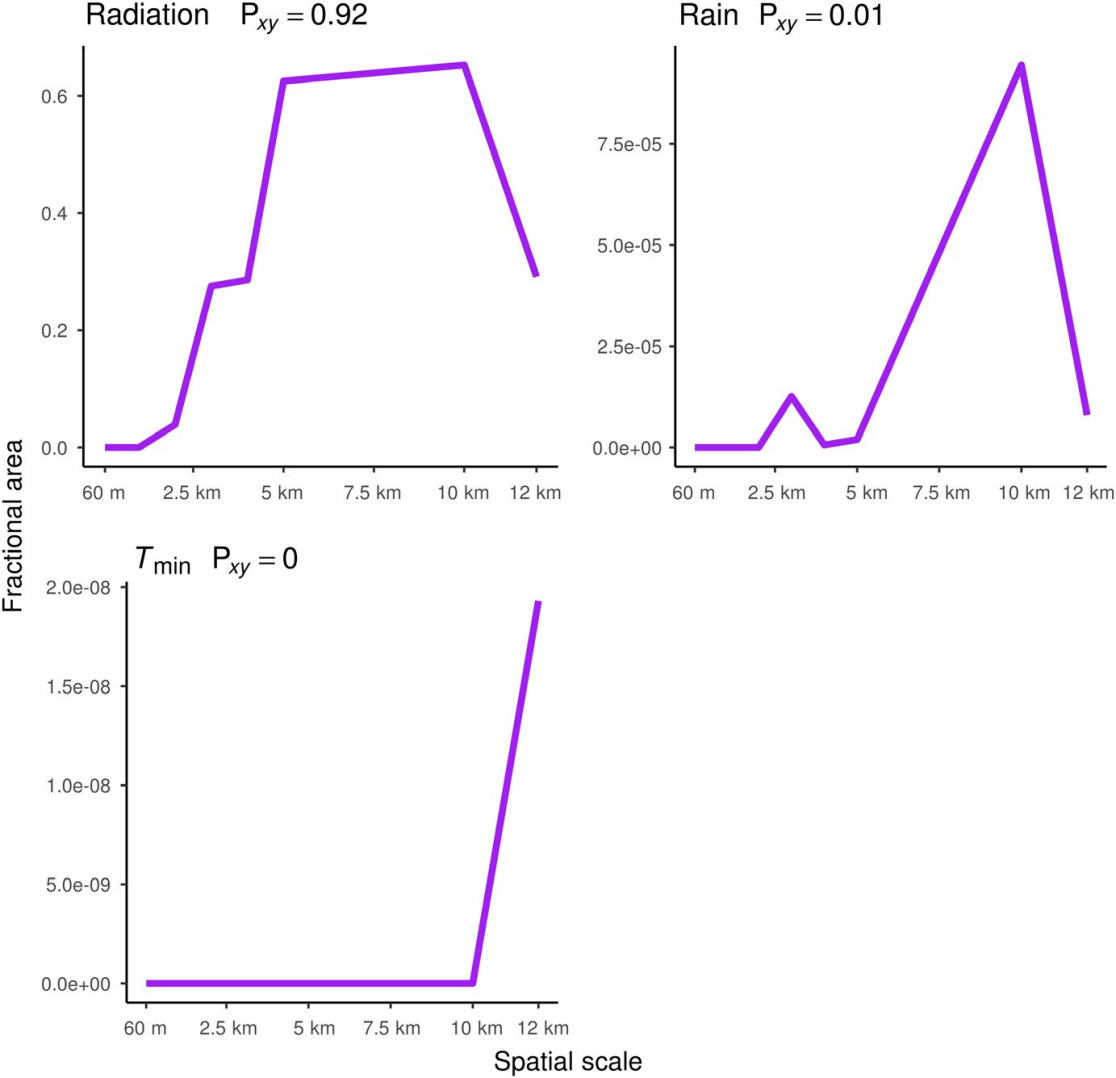
“Both” climate versus spatial scale plots. Spatial scale (60 m–12 km) plotted against fractional area of “both” climates for each climate variable. The Spearman's rank correlation coefficient is included in each plot

The relationship between spatial resolution and fractional area of “both” novel and disappeared climates at the same locations was less clear than novel, disappeared, and shared climates (Figure 10). While “both” climates were virtually nonexistent at 60‐m resolution for all climate surfaces except *T*_min_ climates, the occurrence of “both” climates noticeably increased for rain and radiation climate surfaces at coarser resolutions. *T*_min_ climate surfaces did not have any noticeable increase in both novel and disappeared *T*_min_ climates occurring in the same location at any resolution. “Both” climates for radiation displayed a very strong Spearman's rank correlation between spatial scale and fractional area, indicating that as radiation climate becomes coarser, the amount of both novel and disappeared climates at the same location will increase. However, rain and *T*_min_ had extremely low correlations, indicating that an increased occurrence of both novel and disappeared climates at the same will not always occur as climate resolution becomes coarser (Figure 10).

## 4 DISCUSSION

As climate changes, either from natural processes and/or human activity, novel and disappeared climates will arise (Jackson & Overpeck, 2000; Williams & Jackson, 2007). Long‐term climate patterns that affect observed novel and disappeared climates, whether in the past, present, or future, arise not only from geographic and temporal changes in the environment, but also as a result of atmospheric and topographic processes that occur across many spatial scales (Ackerly et al., 2010).

Determining how climate resolution affects climate change predictions is necessary to avoid under or overestimating the potential impacts of climate change for ecological analyses dependent on gridded climate data. We downscaled climate data at 11 spatial resolutions spanning three orders of magnitude to include climate data at resolutions used for previous climate change impact projections to identify the distribution and abundance of novel, disappeared, and shared climates across Alaska, as well as how the spatial resolution of climate data used affects these estimates of climate (e.g., PRISM, BioClim, NASA Nex, CMIP; Daly et al., 2004; Fick & Hijmans, 2017; Thrasher et al., 2013). We found that novel and disappeared climates primarily affected Southern Alaska. Additionally, climate data generally increased the fractional area of novel and disappeared climates as the resolution became coarser; however, it was also possible, although rare, to decrease the fractional area or have no apparent relationship between the fractional area of novel and disappeared climates and climate data resolution.

### 4.1 Novel, disappeared, and shared climate distributions

All our downscaled climate surfaces, except rain, contained disappeared climates across Alaska. Rain was the only surface that did not contain disappeared climate because (a) rain values cannot reach lower than 0 mm of precipitation (unlike temperature), and (b) environmentally, warm modern climates produce more rain and less snow, which can only extend the upper limit of possible modern rain values.

Therefore, the combination of these two effects produced no unique rain values for our LGM climate surfaces. Overall, disappeared climates primarily affected the southeastern Coast Mountain and Pacific Mountain Transition ecoregions, where modern climate has significantly warmed causing the extremely low maximum temperature climates of the LGM to disappear. Disappeared snow climates primarily affected the Southern Coastal Rainforest ecoregion, where more precipitation now falls as rain, as opposed to snow during the LGM, causing high levels of LGM snow climates to disappear for the modern era. While the fractional area of disappeared climates in Western Alaska was not as high as Southern Alaska, the Aleutian Meadows and Bering Taiga ecoregions experienced some disappeared AET and PET climates, likely as a result of higher radiation climates during the LGM than the modern era. There is some evidence from paleoclimate simulations that suggests less atmospheric water vapor reduced the amount of low‐level clouds and increased the amount of high‐level clouds, which could result in increased surface radiation while still producing a cooling effect over arctic regions during the LGM (Webb, Rind, Lehman, Healy, & Sigman, 1997).

Only three of our eight downscaled climate surfaces contained novel climate distributions in Alaska. These surfaces included radiation, rain, and minimum temperature; however, novel climates were a rarer occurrence than disappeared climates. Less novel climates, when compared to disappeared climates, are likely an artifact of possible climate ranges of Alaska, since we considered only Alaska as our reference area of interest for this study, which ignores all other possible climate ranges across Earth. Additionally, as with disappeared rain climates, novel rain, snow, radiation, AET, PET, and water deficit climates are environmentally restricted. Therefore, due to natural postglacial and/or anthropogenic climate change, only the southernmost portion of Alaska could experience significant amounts of novel climates. Modern climate change has led to increasingly warmer minimum temperatures in the southern Aleutian Meadows and Coastal Rainforest ecoregions. Consequently, this has eliminated extremely cold minimum temperatures that were present during the LGM, while simultaneously increasing the amount of rainfall in the state due to more precipitation being converted to rain over snow. Additionally, these ecoregions are along the southernmost portions of the state, which are lowest in latitude and should therefore be warmer than any other region of Alaska.

Since novel and disappeared climates identify areas most affected by climate change, shared climates fundamentally capture regions that have been least affected by postglacial climate change. Novel and disappeared climates largely affected Southern Alaska, resulting in lower fractional areas of shared climates in the south, and higher fractional areas in the North. As expected, the most northern ecoregions of the state, the Arctic Tundra, Bering Tundra, and Intermontane Boreal ecoregions, contained the largest amount of shared climates, while the southernmost ecoregions, the Coast and Pacific Mountain Transition ecoregions, contained the least amount of shared climates.

The occurrence of both novel and disappeared climates existing at the same location on our downscaled climate surfaces was virtually nonexistent. *T*_min_ contained a few pixels (11) classified as both novel and disappeared; however, this phenomenon was deemed insignificant because it covered such a small fraction of Alaska's area. At larger spatial resolutions, there are instances where some climate variables have a substantial increase in both novel and disappeared climates occurring at the same location. However, areas where both novel and disappeared climates occurred at the same location at resolutions >60 m were not considered for analysis on the distribution of novel and disappeared climates. Novel and disappeared climates are an important characterization of climate change that help identify the vulnerability of ecoregions, since they can push species beyond their ideal climate space and lead to ecologically risky scenarios such as migration, range shifts, and extinction. The Aleutian Meadows and Coastal Rainforests of Alaska stood out as the most vulnerable ecoregions to postglacial climate change. These two regions generally experienced both large amounts of novel and disappeared climates across all climate variables when compared to all other ecoregions in Alaska. Ecoregions with large shared climate distributions may have important implications for identifying glacial refugia because they represent regions that have experienced the least climate change through time and contain the most stable climates in Alaska. The Bering Tundra was the least vulnerable Alaskan ecoregion to postglacial climate change, as all variables in the region contained extremely low fractional areas of novel or disappeared climates.

While we have identified the least and most climatically vulnerable ecoregions to postglacial climate change, large variations in the abundance and distribution of novel and disappeared climates will also affect the significance of each climate variable on a refugia as specified by the optimal niche species. Situations arose where one to few climate variables contained substantial amounts of novel or disappeared climates, while many others contained low to no novel or disappeared climates. This is important for two reasons: First, if an ecoregion contained significant fractional areas of novel or disappeared climates, it does not necessarily imply that all climate variables within the same ecoregion experienced high amounts of novel or disappeared climates. For instance, the Coast Mountain Transition ecoregion in Alaska contained the largest fractional area of disappeared *T*_Max_ climates. Most other climate variables within the same ecoregion contained low amounts of disappeared climates (Figure 4).

Second, large fractional areas of novel or disappeared climate do not necessarily equate to ecological significance for a species (e.g., niche theory). For example, a species may have a large temperature threshold (generalist) with a low snow threshold (specialist). Even with the significant warming of the LGM (85.0% disappeared *T*_max_), *T*_max_ climates may still fall within the optimal range of the species. However, while changes to LGM snow climates were low (2.3% disappeared snow), this change may be ecologically risky for the species if the changes fall outside its optimal snow range. In this case, disappeared snow climates are significant to a species, not disappeared *T*_max_ climates, even though there was a far greater fractional area of disappeared *T*_max_ climates. This suggests that disappeared or novel climates may only be important if they are a limiting factor or extend beyond the optimal niche for a species (De Baar, 1994; Vandermeer, 1972). If the change in climate is a limiting factor, the risk of distribution shifts or extirpation may increase for a species. However, if the change in climate is not limiting, the existence of novel and disappeared climates may have minimal impacts on a species.

### 4.2 Climate grid resolution and fractional area patterns

**Figure 11:**
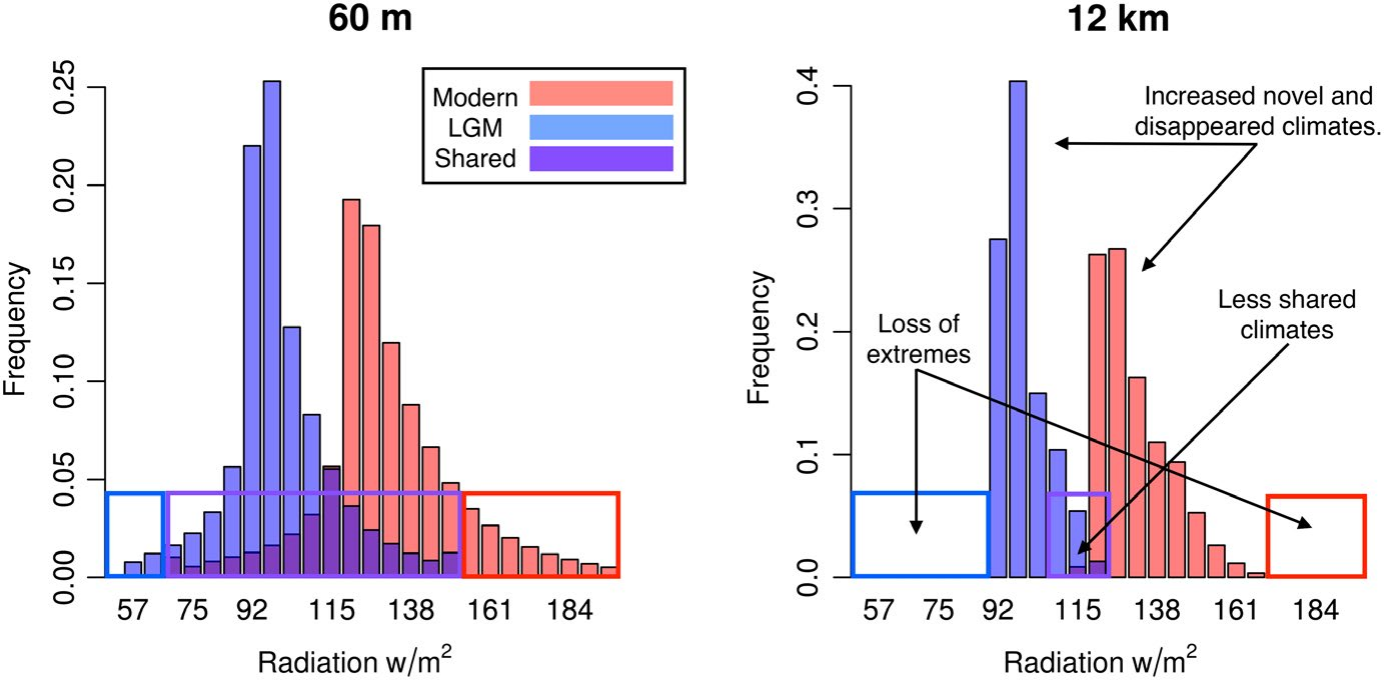
Coarser resolutions can increase the amount of novel and disappeared climates in Alaska. Modern climate ranges are red bars, LGM climate ranges are blue bars, and the overlap of modern and LGM climates are dark blue bars. The red boxes are novel climate bins, blue boxes are disappeared climate bins, and purple boxes are shared climate bins

In our study, the amount of novel and disappeared climate distributions was affected by climate grid resolution in two primary ways. First, as climate data becomes coarser, there is a positive relationship between the fractional area of novel and disappeared climates and spatial resolution. This pattern occurs for two reasons: (a) Coarser climate data reduced environmental variability, which in turn removes the extreme ranges of climate, and (b) there are fewer climates that fall within the shared climate bin definition, resulting in fewer climates classified as shared while simultaneously increasing the frequency of novel or disappeared climates simply due to the coarser scales (Figure 11). As expected, all shared climates displayed a negative relationship between climate resolution and fractional area (Figure 9). Shared climates should display a negative relationship because as the fractional area of novel and disappeared climates increases with spatial resolution, the fractional area of shared climates must decrease because it accounts for areas not classified as either novel or disappeared. Previous studies by Franklin et al. (2013), Heikkinen, Luoto, Kuussaari, and Toivonen (2007), and Seo et al. (2009) found similar results modeling species distributions, finding that coarse climate data predicts larger suitable habitats compared to finer resolution climate data. Our study confirms this trend, although our analysis is focused solely on the distribution of climate as opposed to the distribution of species.

The positive relationship between spatial resolution and novel and disappeared climate distributions suggests that the use of coarse grid climate data may have led to the overestimation of novel and disappeared climate distributions in previous analyses, and thereby cause overestimates of potential impacts of climate change. This may lead to significant errors when trying to determine where postglacial climate change has occurred. Additionally, this error can be propagated into other analyses that depend on climate grid data, such as species distribution models, which implies that these studies may also over or underestimate predicted species ranges, refugial locations, and other climate‐species analyses (Franklin et al., 2013; Randin et al., 2009; Trivedi, Berry, Morecroft, & Dawson, 2008).

**Figure 12:**
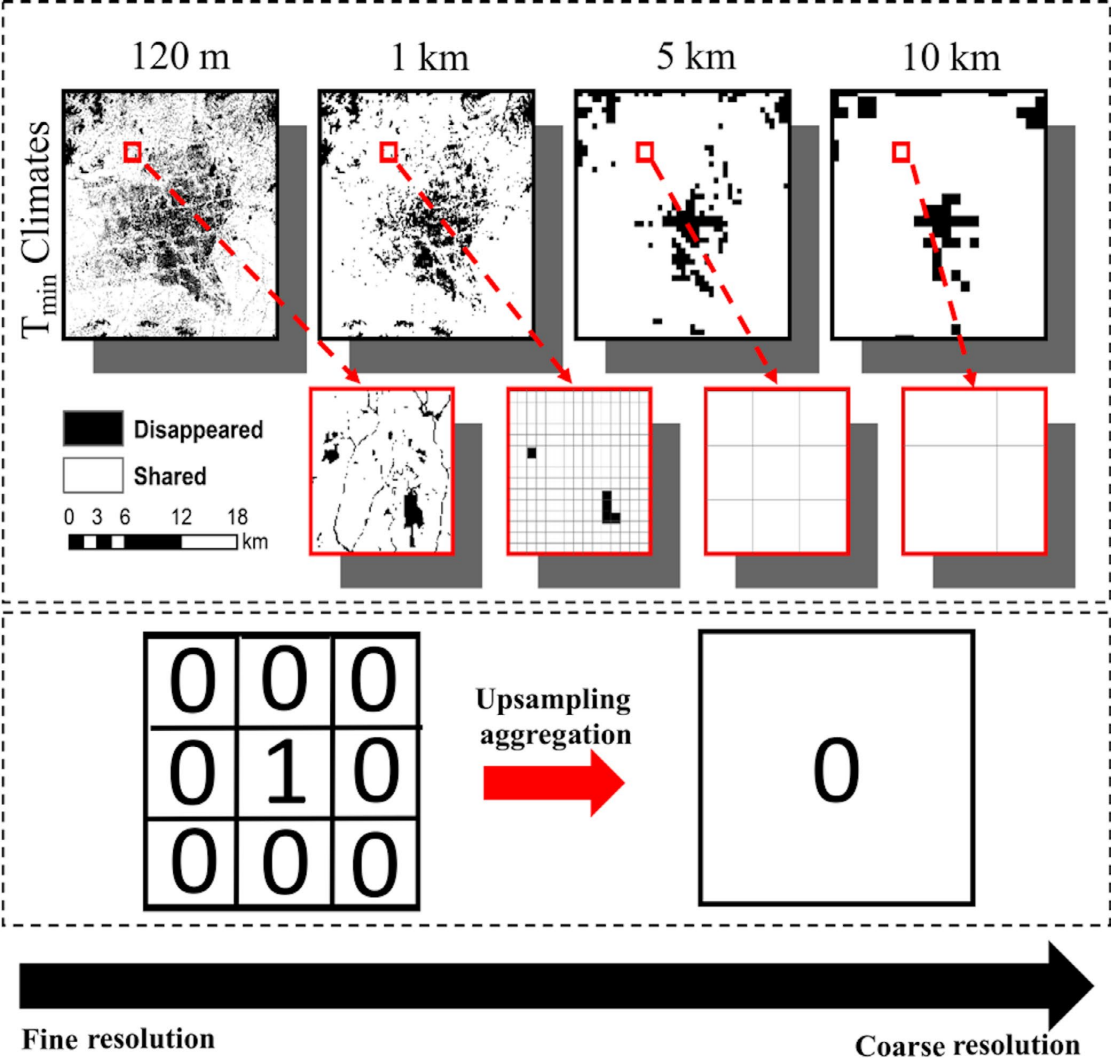
Coarser resolutions can decrease the amount of novel and disappeared climates in Alaska. Disappeared *T*_min_ at 120 m displays regions with patchy, but random spatial distributions, while coarse disappeared *T*_min_ climates display clustered distributions. Isolated patches of disappeared *T*_min_ climates at high resolutions will be reclassified to shared *T*_min_ climates when upsampling *T*_min_ climates to coarser scales using standard pixel aggregation methods

Second, as climate data becomes coarser, there can be a negative relationship between spatial resolution and the amount of novel and disappeared climates, although this trend only occurred with our *T*_min_ disappeared climate surfaces. This pattern can occur when novel or disappeared climates are extremely spatially heterogeneous (i.e., patchy; Figure 12). At coarser resolutions, our *T*_min_ climate aggregation scheme successively grouped similar temperatures together at each progressively coarser resolution (Wu, 1999). Because our 60 m disappeared *T*_min_ surface was extremely patchy, successively aggregating the surface to coarser resolutions averaged out the few pixels classified as disappeared *T*_min_ climates and progressively reclassified coarser *T*_min_ disappeared climates to *T*_min_ shared climates. Randin et al. (2009) reported a similar negative pattern when comparing the distribution of alpine species; however, Randin's study considered climate resolution with varying study extents. The combination of climate resolution and study extent was found to cause a negative relationship between the amount of suitable alpine species habitats and climate resolution/extent as their species distribution models were unable to capture the full environmental niche of alpine species at regional scale climate resolutions or limited study extents. Therefore, the negative pattern between alpine species distributions and climate data resolution from Randin et al. (2009) was not a product of heterogeneously complex climate surfaces.

Although a negative relationship between fractional area and spatial resolution occurred on only one climate surface, this phenomenon suggests it is possible to underestimate the amount of novel and disappeared climates when using coarse gridded climate products. Again, significant errors may arise when estimating postglacial climate change distributions leading to serious underestimations of novel and disappeared climate distributions, the potential impacts of climate change, and potentially propagating this error into other climate dependent analyses. However, this pattern is less likely to occur since it depends on the patchiness of novel and disappeared climates as opposed to gridded climate data's ability to capture landscape scale processes influencing climate patterns.

No significant relationship between fractional area of novel and disappeared climates and spatial resolution is possible, and this pattern did not visibly arise within our study. There were some cases where the Spearman's rank correlation was “moderate”, although this does not necessarily indicate a relationship. However, while all surfaces displayed moderate to strong correlations, there was a fair amount of variation in fractional area as spatial resolution became coarser. This suggests that there may be significant thresholds occurring at various spatial scales. The exact causes of this variation are still unclear, but we hypothesize that this variation may be a result of distortion caused by aliasing artifacts when upsampling our 60 m downscaled climate surfaces to coarser scales (Haines‐Young & Chopping, 1996; Kennie & McLaren, 1988), or may be caused by Moiré fringes (Gustafsson, 2000) producing overlooked oscillations across a landscape. While Moiré fringes are a well‐known issue affecting raster image processing and visualization, to the best of our knowledge, no studies have investigated whether naturally occurring processes and phenomenon known to influence climate or other Earth systems, generate spatial Moiré distributions.

### 4.3 Limitations

Annual averages are a limitation because summer temperatures are not so different from today; however, the rest of the year was, so annual averages do not capture the seasonal climate variation over Alaska. This is only an issue in terms of when we are talking about the interpretation of novel and disappeared climates impact on plants, not on how scale affects the amount of novel and disappeared climates predicted since seasonality does not affect the definition of a novel or disappeared climate. Future studies should look into seasonal variation of novel and disappeared climates.

## 5 CONCLUSIONS

It is important to understand how climate change through time affects the distribution and abundance of novel and disappeared climates, as well as how choice in gridded climate data resolution affects our estimations of climate change. If our 60 m downscaled climate surfaces accurately reflect physiographic processes affecting climate at multiple scales, our fine‐scale climate products should improve estimations of novel and disappeared climate distributions, compared to coarser climate products, especially in areas with high topographic complexity.

## CONFLICT OF INTEREST

None declared.

## AUTHOR CONTRIBUTIONS

JAG and KH conceived the presented analysis and interpretation of data and revised the draft manuscript. JAG supervised the methods and findings of the work. BDM acquired data, developed the models, performed the analysis, and drafted the manuscript of the presented research.

## Data Availability

Downscaled climate data output files: ORNL DAAC https://doi.org/10.3334/ORNLDAAC/1663.

## References

[ece35511-bib-0001] Ackerly, D. D. , Loarie, S. R. , Cornwell, W. K. , Weiss, S. B. , Hamilton, H. , Branciforte, R. , & Kraft, N. J. B. (2010). The geography of climate change: Implications for conservation biogeography. Diversity and Distributions, 16, 476–487. 10.1111/j.1472-4642.2010.00654.x

[ece35511-bib-0002] Aggarwal, C. C. (2016). Outlier analysis, 2nd ed. Cham, Switzerland: Springer 10.1007/978-3-319-47578-3

[ece35511-bib-0003] Allen, R. G. , Pereira, L. S. , Raes, D. , & Smith, M. (1998). Crop evapotranspiration‐Guidelines for computing crop water requirements‐FAO Irrigation and drainage paper 56. Irrigation and Drainage.

[ece35511-bib-0004] Bellard, C. , Bertelsmeier, C. , Leadley, P. , Thuiller, W. , & Courchamp, F. (2012). Impacts of climate change on the future of biodiversity. Ecology Letters, 15, 365–377. 10.1111/j.1461-0248.2011.01736.x 22257223 PMC3880584

[ece35511-bib-0005] Berryman, A. A. (2003). On principles, laws and theory in population ecology. Oikos, 103, 695–701. 10.1034/j.1600-0706.2003.12810.x

[ece35511-bib-0006] Breiman, L. (2001). Random forests. Machine Learning, 45, 5–32.

[ece35511-bib-0007] Burrows, M. T. , Schoeman, D. S. , Richardson, A. J. , Molinos, J. G. , Hoffmann, A. , Buckley, L. B. , … Poloczanska, E. S. (2014). Geographical limits to species‐range shifts are suggested by climate velocity. Nature, 507, 492 10.1038/nature12976 24509712

[ece35511-bib-0008] Cannon, A. J. (2011). Quantile regression neural networks: Implementation in R and application to precipitation downscaling. Computers & Geosciences, 37, 1277–1284. 10.1016/j.cageo.2010.07.005

[ece35511-bib-0009] Chen, I. C. , Hill, J. K. , Ohlemuller, R. , Roy, D. B. , & Thomas, C. D. (2011). Rapid range shifts of species associated with high levels of climate warming. Science, 333, 1024–1026. 10.1126/science.1206432 21852500

[ece35511-bib-0010] Crimmins, S. M. , Dobrowski, S. Z. , Greenberg, J. A. , Abatzoglou, J. T. , & Mynsberge, A. R. (2011). Changes in climatic water balance drive downhill shifts in plant species' optimum elevations. Science, 331, 324–327. 10.1126/science.1199040 21252344

[ece35511-bib-0011] Daly, C. (2006). Guidelines for assessing the suitability of spatial climate data sets. International Journal of Climatology, 26, 707–721. 10.1002/joc.1322

[ece35511-bib-0012] Daly, C. , Gibson, W. , Doggett, M. , Smith, J. , & Taylor, G. (2004). Up‐to‐date monthly climate maps for the conterminous United States In Proc., 14th AMS Conf. on Applied Climatology, 84th AMS Annual Meeting Combined Preprints, Amer. Meteorological Soc., Seattle, WA.

[ece35511-bib-0013] Davis, M. B. (1990). Climatic change and the survival of forest species In Woodwell G. M. (Ed.), The earth in transition: Patterns and processes of biotic impoverishment (pp. 99–111). Cambridge, UK: Cambridge University Press.

[ece35511-bib-0014] De Baar, H. (1994). von Liebig's law of the minimum and plankton ecology (1899–1991). Progress in Oceanography, 33, 347–386. 10.1016/0079-6611(94)90022-1

[ece35511-bib-0016] Dobrowski, S. Z. , Abatzoglou, J. T. , Greenberg, J. A. , & Schladow, S. G. (2009). How much influence does landscape‐scale physiography have on air temperature in a mountain environment? Agricultural and Forest Meteorology, 149, 1751–1758. 10.1016/j.agrformet.2009.06.006

[ece35511-bib-0017] Dobrowski, S. Z. , Abatzoglou, J. , Swanson, A. K. , Greenberg, J. A. , Mynsberge, A. R. , Holden, Z. A. , & Schwartz, M. K. (2013). The climate velocity of the contiguous United States during the 20th century. Global Change Biology, 19, 241–251. 10.1111/gcb.12026 23504735

[ece35511-bib-0018] Dunne, K. , & Willmott, C. J. (1996). Global distribution of plant‐extractable water capacity of soil. International Journal of Climatology, 16, 841–859. 10.1002/(SICI)1097-0088(199608)16:8<841:AID-JOC60>3.0.CO;2-8

[ece35511-bib-0019] Fick, S. E. , & Hijmans, R. J. (2017). WorldClim 2: New 1‐km spatial resolution climate surfaces for global land areas. International Journal of Climatology, 37, 4302–4315. 10.1002/joc.5086

[ece35511-bib-0020] Fitzpatrick, M. C. , & Hargrove, W. W. (2009). The projection of species distribution models and the problem of non‐analog climate. Biodiversity and Conservation, 18, 2255–2261. 10.1007/s10531-009-9584-8

[ece35511-bib-0021] Ford, J. D. , Keskitalo, E. , Smith, T. , Pearce, T. , Berrang‐Ford, L. , Duerden, F. , & Smit, B. (2010). Case study and analogue methodologies in climate change vulnerability research. Wiley Interdisciplinary Reviews: Climate Change, 1, 374–392. 10.1002/wcc.48

[ece35511-bib-0022] Fordham, D. A. , Saltré, F. , Brown, S. C. , Mellin, C. , & Wigley, T. M. (2018). Why decadal to century timescale palaeoclimate data are needed to explain present‐day patterns of biological diversity and change. Global Change Biology, 24, 1371–1381. 10.1111/gcb.13932 28994170

[ece35511-bib-0023] Franklin, J. , Davis, F. W. , Ikegami, M. , Syphard, A. D. , Flint, L. E. , Flint, A. L. , & Hannah, L. (2013). Modeling plant species distributions under future climates: How fine scale do climate projections need to be? Global Change Biology, 19, 473–483. 10.1111/gcb.12051 23504785

[ece35511-bib-0024] Gallant, A. L. , Binnian, E. F. , Omernik, J. M. , & Shasby, M. B. (1995). Ecoregions of Alaska. Washington, DC: US Government Printing Office.

[ece35511-bib-0025] Geiger, R. , Aron, R. H. , & Todhunter, P. (2009). The climate near the ground. Lanham, MD: Rowman & Littlefield.

[ece35511-bib-0026] Gent, P. R. , Danabasoglu, G. , Donner, L. J. , Holland, M. M. , Hunke, E. C. , Jayne, S. R. , … Zhang, M. (2011). The Community Climate System Model Version 4. Journal of Climate, 24, 4973–4991. 10.1175/2011JCLI4083.1

[ece35511-bib-0027] Glassberg, D. (2014). Place, memory, and climate change. Public Historian, 36, 17–30. 10.1525/tph.2014.36.3.17 25638963

[ece35511-bib-0028] Grenier, P. , Parent, A.‐C. , Huard, D. , Anctil, F. , & Chaumont, D. (2013). An assessment of six dissimilarity metrics for climate analogs. Journal of Applied Meteorology and Climatology, 52, 733–752. 10.1175/JAMC-D-12-0170.1

[ece35511-bib-0029] Gustafsson, M. G. (2000). Surpassing the lateral resolution limit by a factor of two using structured illumination microscopy. Journal of Microscopy, 198, 82–87. 10810003 10.1046/j.1365-2818.2000.00710.x

[ece35511-bib-0030] Haines‐Young, R. , & Chopping, M. (1996). Quantifying landscape structure: A review of landscape indices and their application to forested landscapes. Progress in Physical Geography, 20, 418–445. 10.1177/030913339602000403

[ece35511-bib-0031] Heikkinen, R. K. , Luoto, M. , Kuussaari, M. , & Toivonen, T. (2007). Modelling the spatial distribution of a threatened butterfly: Impacts of scale and statistical technique. Landscape and Urban Planning, 79, 347–357. 10.1016/j.landurbplan.2006.04.002

[ece35511-bib-0032] Hobbs, R. J. , Arico, S. , Aronson, J. , Baron, J. S. , Bridgewater, P. , Cramer, V. A. , … Zobel, M. (2006). Novel ecosystems: Theoretical and management aspects of the new ecological world order. Global Ecology and Biogeography, 15, 1–7. 10.1111/j.1466-822X.2006.00212.x

[ece35511-bib-0035] Hofierka, J. , & Suri, M. (2002). The solar radiation model for open source GIS: Implementation and applications. Proceedings of the open source GIS-GRASS users conference, 2002, 51–70.

[ece35511-bib-0033] Hutchinson, G. (1957). Concluding remarks Cold Spring Harbor Symp. Quant. 22, 66–77.

[ece35511-bib-0034] Jackson, S. T. , & Overpeck, J. T. (2000). Responses of plant populations and communities to environmental changes of the late Quaternary. Paleobiology, 26, 194–220. 10.1017/S0094837300026932

[ece35511-bib-0036] Katurji, M. , & Zhong, S. Y. (2012). The influence of topography and ambient stability on the characteristics of cold‐air pools: A numerical investigation. Journal of Applied Meteorology and Climatology, 51, 1740–1749. 10.1175/JAMC-D-11-0169.1

[ece35511-bib-0037] Kennie, T. , & Mclaren, R. (1988). Modelling for digital terrain and landscape visualisation. The Photogrammetric Record, 12, 711–745. 10.1111/j.1477-9730.1988.tb00626.x

[ece35511-bib-0038] Kluzek, E. (2011). CCSM research tools: CLM4. 0 user's guide documentation.

[ece35511-bib-0039] Larsen, J. N. , Anisimov, O. A. , Constable, A. , Hollowed, A. B. , Maynard, N. , Prestrud, P. , … Wrona, F. (2014). Polar regions In Barros V. R., Field C. B., Dokken D. J., Mastrandrea M. D., Mach K. J., Bilir T. E., Chatterjee M., Ebi K. L., Estrada Y. O., Genova R. C., Girma B., Kissel E. S., Levy A. N., MacCracken S., Mastrandrea P. R., & White L. L. (Eds.), Climate change 2014: Impacts, adaptation, and vulnerability. Part B: Regional aspects. Contribution of working group II to the fifth assessment report of the intergovernmental panel on climate change. Cambridge, UK: Cambridge University Press.

[ece35511-bib-0040] Levin, S. A. (1992). The problem of pattern and scale in ecology. Ecology, 73, 1943–1967.

[ece35511-bib-0041] Loarie, S. R. , Duffy, P. B. , Hamilton, H. , Asner, G. P. , Field, C. B. , & Ackerly, D. D. (2009). The velocity of climate change. Nature, 462, 1052–1055. 10.1038/nature08649 20033047

[ece35511-bib-0042] Lutz, J. A. , Van Wagtendonk, J. W. , & Franklin, J. F. (2010). Climatic water deficit, tree species ranges, and climate change in Yosemite National Park. Journal of Biogeography, 37(5), 936–950.

[ece35511-bib-0043] Madsen, H. , & Thyregod, P. (2010). Introduction to general and generalized linear models. Boca Raton, FL: CRC Press.

[ece35511-bib-0044] Maraun, D. , Wetterhall, F. , Ireson, A. M. , Chandler, R. E. , Kendon, E. J. , Widmann, M. , … Thiele‐Eich, I. (2010). Precipitation downscaling under climate change: Recent developments to bridge the gap between dynamical models and the end user. Reviews of Geophysics, 48 10.1029/2009RG000314

[ece35511-bib-0045] Mearns, L. , Hulme, M. , Carter, T. , Leemans, R. , Lal, M. , & Whetton, P. (2001). Climate scenario development Climate change 2001: The scientific basis. Contribution of Working. Group I to the Third Assessment Report of the Intergovernmental Panel on Climate Change. Cambridge, UK: Cambridge University Press.

[ece35511-bib-0046] Menne, M. J. , Durre, I. , Vose, R. S. , Gleason, B. E. , & Houston, T. G. (2012). An overview of the global historical climatology network‐daily database. Journal of Atmospheric and Oceanic Technology, 29, 897–910. 10.1175/JTECH-D-11-00103.1

[ece35511-bib-0047] Mix, A. C. , Bard, E. , & Schneider, R. (2001). Environmental processes of the ice age: Land, oceans, glaciers (EPILOG). Quaternary Science Reviews, 20, 627–657. 10.1016/S0277-3791(00)00145-1

[ece35511-bib-0048] Monteith, J. L. (1965). Evaporation and environment, in the state and movement of water in living organisms In Fogg G. E. (Ed.), Symp. Soc. Exp. Biol (pp. 205–234). New York, NY: Academic Press. 5321565

[ece35511-bib-0049] Ordonez, A. , Williams, J. W. , & Svenning, J.‐C. (2016). Mapping climatic mechanisms likely to favour the emergence of novel communities. Nature Climate Change, 6, 1104 10.1038/nclimate3127

[ece35511-bib-0050] Overpeck, J. T. , Bartlein, P. J. , & Webb, T. (1991). Potential magnitude of future vegetation change in eastern North America: Comparisons with the past. Science, 254, 692–695. 10.1126/science.254.5032.692 17774796

[ece35511-bib-0051] Penman, H. L. (1948). Natural evaporation from open water, bare soil and grass In Proceedings of the Royal Society of London A: Mathematical, Physical and Engineering Sciences. London, UK: The Royal Society. 10.1098/rspa.1948.0037 18865817

[ece35511-bib-0052] Radeloff, V. C. , Williams, J. W. , Bateman, B. L. , Burke, K. D. , Carter, S. K. , Childress, E. S. , … Usinowicz, J. (2015). The rise of novelty in ecosystems. Ecological Applications, 25, 2051–2068. 10.1890/14-1781.1 26910939

[ece35511-bib-0053] Raisanen, J. (2001). CO2‐induced climate change in CMIP2 experiments: Quantification of agreement and role of internal variability. Journal of Climate, 14, 2088–2104.

[ece35511-bib-0054] Randin, C. F. , Engler, R. , Normand, S. , Zappa, M. , Zimmermann, N. E. , Pearman, P. B. , … Guisan, A. (2009). Climate change and plant distribution: Local models predict high‐elevation persistence. Global Change Biology, 15, 1557–1569. 10.1111/j.1365-2486.2008.01766.x

[ece35511-bib-0055] Ruiz‐Arias, J. , Tovar‐Pescador, J. , Pozo‐Vázquez, D. , & Alsamamra, H. (2009). A comparative analysis of DEM‐based models to estimate the solar radiation in mountainous terrain. International Journal of Geographical Information Science, 23, 1049–1076. 10.1080/13658810802022806

[ece35511-bib-0056] Seo, C. , Thorne, J. H. , Hannah, L. , & Thuiller, W. (2009). Scale effects in species distribution models: Implications for conservation planning under climate change. Biology Letters, 5, 39–43. 10.1098/rsbl.2008.0476 18986960 PMC2657743

[ece35511-bib-0057] Stephenson, N. L. (1990). Climatic control of vegetation distribution – the role of the water‐balance. The American Naturalist, 135, 649–670. 10.1086/285067

[ece35511-bib-0058] Stephenson, N. L. (1998). Actual evapotranspiration and deficit: Biologically meaningful correlates of vegetation distribution across spatial scales. Journal of Biogeography, 25, 855–870. 10.1046/j.1365-2699.1998.00233.x

[ece35511-bib-0059] Suri, M. , & Hofierka, J. (2004). A new GIS‐based solar radiation model and its application to photovoltaic assessments. Transactions in GIS, 8, 15 10.1111/j.1467-9671.2004.00174.x

[ece35511-bib-0060] Taylor, K. E. , Stouffer, R. J. , & Meehl, G. A. (2012). An overview of CMIP5 and the experiment design. Bulletin of the American Meteorological Society, 93, 485–498. 10.1175/BAMS-D-11-00094.1

[ece35511-bib-0061] Tesfa, T. K. , Tarboton, D. G. , Watson, D. W. , KaT, S. , Baker, M. E. , & Wallace, R. M. (2011). Extraction of hydrological proximity measures from DEMs using parallel processing. Environmental Modelling & Software, 26, 1696–1709. 10.1016/j.envsoft.2011.07.018

[ece35511-bib-0062] Thrasher, B. , Xiong, J. , Wang, W. , Melton, F. , Michaelis, A. , & Nemani, R. (2013). Downscaled climate projections suitable for resource management. Eos, Transactions American Geophysical Union, 94, 321–323. 10.1002/2013EO370002

[ece35511-bib-0063] Trivedi, M. R. , Berry, P. M. , Morecroft, M. D. , & Dawson, T. P. (2008). Spatial scale affects bioclimate model projections of climate change impacts on mountain plants. Global Change Biology, 14, 1089–1103. 10.1111/j.1365-2486.2008.01553.x

[ece35511-bib-0064] Urban, D. L. , Miller, C. , Halpin, P. N. , & Stephenson, N. L. (2000). Forest gradient response in Sierran landscapes: The physical template. Landscape Ecology, 15, 603–620.

[ece35511-bib-0065] USGS (2016). The National Map. U. S. G. Survey. 3D Elevation Program Web Page, 3DEP Products and Services.

[ece35511-bib-0066] Vandermeer, J. H. (1972). Niche theory. Annual Review of Ecology and Systematics, 3, 107–132. 10.1146/annurev.es.03.110172.000543

[ece35511-bib-0067] Venables, W. , & Ripley, B. (2002). Modern applied statistics with S. New York, NY: Springer.

[ece35511-bib-0068] Webb III, T. (1992). Past change in vegetation and climate: Lessons for the future In Peters R. L. & Lovejoy T. S. (Eds.), Global warming and biological diversity (pp. 59–75). New Haven, CT: Yale Univerity Press.

[ece35511-bib-0069] Webb, R. S. , Rind, D. H. , Lehman, S. J. , Healy, R. J. , & Sigman, D. (1997). Influence of ocean heat transport on the climate of the Last Glacial Maximum. Nature, 385, 695 10.1038/385695a0

[ece35511-bib-0070] Wetterhall, F. , Bardossy, A. , Chen, D. L. , Halldin, S. , & Xu, C. Y. (2006). Daily precipitation‐downscaling techniques in three Chinese regions. Water Resources Research, 42 10.1029/2005WR004573

[ece35511-bib-0071] Widmann, M. , Bretherton, C. S. , & Salathé, E. P. Jr (2003). Statistical precipitation downscaling over the northwestern United States using numerically simulated precipitation as a predictor. Journal of Climate, 16, 799–816. 10.1175/1520-0442(2003)016<0799:SPDOTN>2.0.CO;2

[ece35511-bib-0072] Wiens, J. A. , Seavy, N. E. , & Jongsomjit, D. (2011). Protected areas in climate space: What will the future bring? Biological Conservation, 144, 2119–2125. 10.1016/j.biocon.2011.05.002

[ece35511-bib-0073] Williams, J. W. , & Jackson, S. T. (2007). Novel climates, no‐analog communities, and ecological surprises. Frontiers in Ecology and the Environment, 5, 475–482. 10.1890/070037

[ece35511-bib-0074] Williams, J. W. , Jackson, S. T. , & Kutzbacht, J. E. (2007). Projected distributions of novel and disappearing climates by 2100 AD. Proceedings of the National Academy of Sciences of the United States of America, 104(14), 5738–5742. 17389402 10.1073/pnas.0606292104 PMC1851561

[ece35511-bib-0075] Willis, K. J. , & Bhagwat, S. A. (2009). Biodiversity and climate change. Science, 326, 806–807. 10.1126/science.1178838 19892969

[ece35511-bib-0076] Wolock, D. M. , & Mccabe, G. J. (1995). Comparison of single and multiple flow direction algorithms for computing topographic parameters in TOPMODEL. Water Resources Research, 31, 1315–1324. 10.1029/95WR00471

[ece35511-bib-0077] Wu, J. (1999). Hierarchy and scaling: Extrapolating information along a scaling ladder. Canadian Journal of Remote Sensing, 25, 367–380. 10.1080/07038992.1999.10874736

